# Visual explanations from spiking neural networks using inter-spike intervals

**DOI:** 10.1038/s41598-021-98448-0

**Published:** 2021-09-24

**Authors:** Youngeun Kim, Priyadarshini Panda

**Affiliations:** Department of Electrical Engineering, Yale University, New Haven, CT USA

**Keywords:** Electrical and electronic engineering, Computational neuroscience, Visual system

## Abstract

By emulating biological features in brain, Spiking Neural Networks (SNNs) offer an energy-efficient alternative to conventional deep learning. To make SNNs ubiquitous, a ‘visual explanation’ technique for analysing and explaining the internal spike behavior of such temporal deep SNNs is crucial. Explaining SNNs visually will make the network more transparent giving the end-user a tool to understand how SNNs make temporal predictions and why they make a certain decision. In this paper, we propose a bio-plausible visual explanation tool for SNNs, called Spike Activation Map (SAM). SAM yields a heatmap (*i*.*e*., localization map) corresponding to each time-step of input data by highlighting neurons with short inter-spike interval activity. Interestingly, without the use of gradients and ground truth, SAM produces a temporal localization map highlighting the region of interest in an image attributed to an SNN’s prediction at each time-step. Overall, SAM outsets the beginning of a new research area ‘*explainable neuromorphic computing*’ that will ultimately allow end-users to establish appropriate trust in predictions from SNNs.

## Introduction

Human brain is the most remarkable neural network. It consists of multiple layers of neurons that re-weight their connection based on the target task. Artificial Neural Networks (ANNs) or conventional deep learning models reasonably emulate the structural feature of the visual cortex and show human-level performance on a wide variety of tasks^1–3^. Nonetheless, ANNs incur huge computational cost to achieve such feats, while an average human brain operates within a power budget of nearly 20 W^4^. Many real-world platforms, such as, smart phones, self-driving cars, voice assistant devices (like Alexa) among others, have resource and battery constraints^5^. To enable intelligence on such platforms, low-power implementation of neural networks is crucial. Spiking Neural Networks (SNNs)^6–11^ offer an alternative and bio-plausible manner for enabling low-power intelligence. SNNs emulate biological neuronal functionality by processing visual information with binary events (*i*.*e*., spikes) over multiple time-steps. This discrete spiking behavior of SNNs has been shown to yield high energy-efficiency on emerging neuromorphic hardware^12–14^.

In the recent past, two broad algorithmic optimization methods for SNNs have made great strides towards bringing the performance of SNNs closer to that of ANNs on image classification (even on the Imagenet dataset that is considered as the ‘Olympics’ among image classification tasks). The first approach, *Conversion*^15–18^, converts a pre-trained ANN to an SNN by normalizing firing thresholds or weights to transfer a ReLU (Rectified Linear Unit) activation to Integrate-and-Fire (IF) spiking activation. So far, conversion techniques have been able to achieve competitive accuracy with ANN counterparts on large-scale architectures and datasets but incur large latency or time-steps for processing. The second approach comprises of surrogate gradient descent methods^16,19,20^ that train SNNs using an approximate gradient function to overcome the non-differentiability of the Leaky-Integrate-and-Fire (LIF) spiking neuron^21^. Such methods enable SNNs to be trained from scratch with lower latency and reasonable classification accuracy.

Despite significant progress in optimization techniques, there is a lack of understanding pertaining to internal spike behavior of SNNs compared to conventional ANN. Neural networks have been conceived to be ‘black-boxes’. However, with ubiquitous usage of neural networks, there is a need to understand what happens when a network predicts or makes a decision. On the ANN front, several interpretation or ‘visual explanation’ tools have been proposed^22–25^. These tools have found practical usage for obtaining visual explanations and understanding the network prediction. On similar lines, an SNN interpretation tool is also highly crucial because low-power SNNs are increasingly becoming viable candidates for deployment in real-world applications such as medical robots^26^, self-driving cars^27^, and drones^28^, where explainability in addition to performance is critical. In this work, we aim to shed light on the explainability of SNNs.

**Figure 1:**
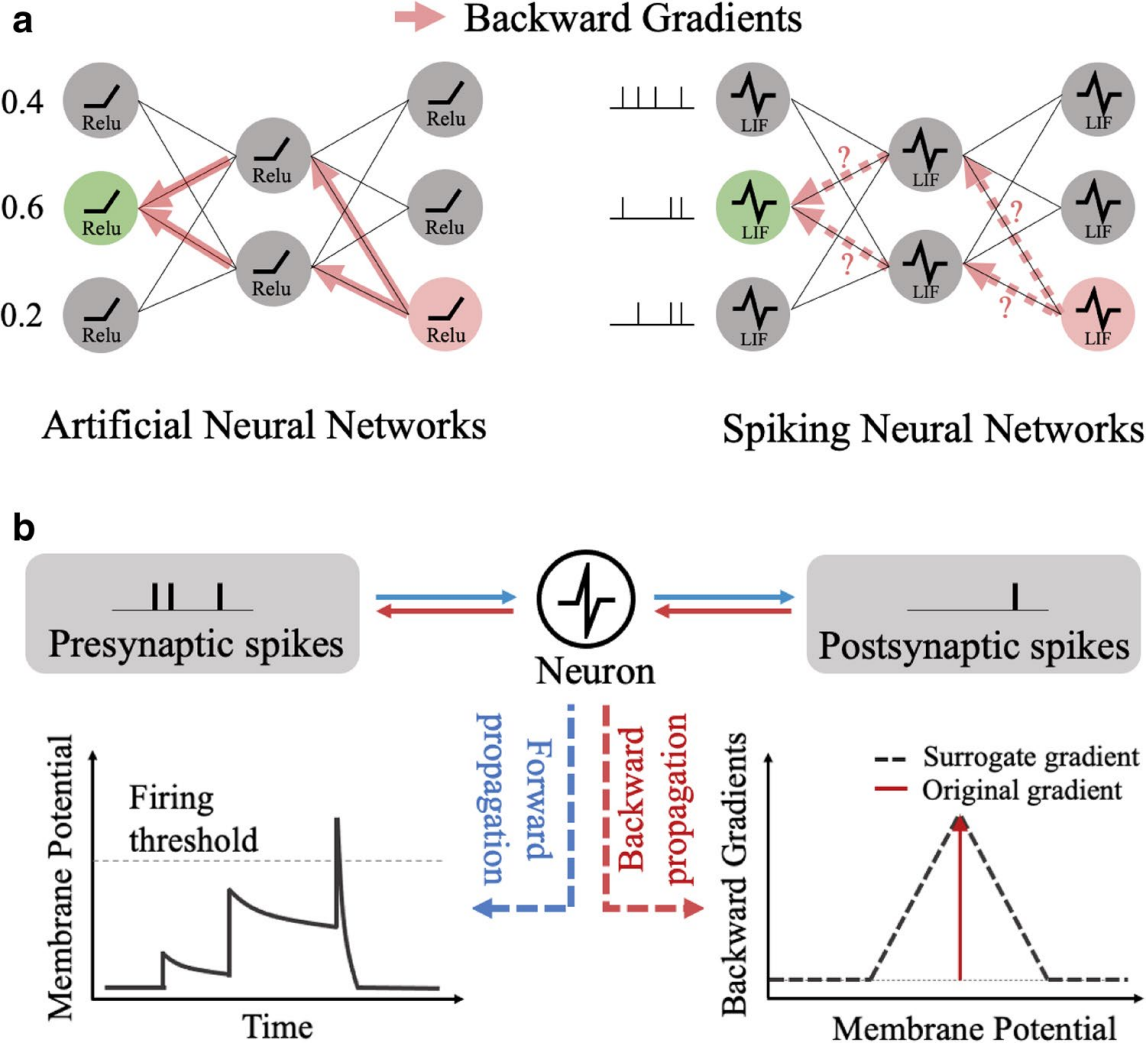
Non-differentiable spiking neural networks. (**a**) Different from ANNs, backward gradients of SNNs are difficult to be calculated. (**b**) The illustration of forward propagation (blue arrow) and backward propagation (red arrow) of an LIF neuron. During forward propagation, the membrane potential increases according to the pre-synaptic spike input. If the membrane potential exceeds the firing threshold, the LIF neuron generates the post-synaptic spike and resets the membrane potential (see “Methods” for details). This leak-integrate-and-fire behavior induces the non-differentiability of the membrane potential. Therefore, surrogate gradient functions are used to implement the backward gradient.

The naïve approach for explainability is to exploit widely used visualization tools from ANN domain. Among them, Grad-CAM^25^ has a huge flexibility in terms of application, and is also used by state-of-the-art interpretation algorithms^29^. The authors of Grad-CAM show that the contribution of a neuron from shallow layers to deep layers towards any target class prediction can be quantified by calculating the gradient with backpropagation. But, SNNs cannot compute exact gradient (*i*.*e*., contribution) because of the non-differentiable integrate and firing behavior of a spiking neuron as shown in Fig. 1. Further, it is hard to imagine how such gradient-based visualization (like Grad-CAM) can be brain-like or can emulate any reasoning capabilities of the brain. First of all, a biological neuron cannot compute exact gradients (*i*.*e*., contribution)^6^. Also, there is no guarantee that the brain holds a precise symmetric copy of the downstream synaptic weight matrix during backpropagation^30,31^. Therefore, a new concept of visualization that takes advantage of the bio-plausible temporal processing in SNNs needs to be explored.

In this study, we propose a visualization tool for SNNs, called Spike Activation Map (SAM). SAM does not require any backpropagation or rely on gradients to obtain ‘visual explanations’. Instead, we calculate a heatmap (or localization map) by monitoring neurons that carry more information (*i*.*e*., spikes) over different time-steps during forward propagation. We leverage the biological observation that short Inter-Spike Interval (ISI) spikes have more information in a neurological system^32–34^ because these spikes are more likely to induce post-synaptic spikes by increasing the membrane potential of a neuron. Given a prediction made by an SNN, SAM computes a *Neuronal Contribution Score* (NCS) for each neuron in the network. The NCS score is defined as the sum of *Temporal Spike Contribution Score* (TSCS) of previous spikes with an exponential kernel. TSCS is high for a neuron that spikes multiple times within a short time window. In contrast, when a neuron fires over a longer period, TSCS is low. Then, we add the NCS values to obtain the heatmap over time that highlights the important regions in the image attributed to the SNN’s prediction. We note that, unlike conventional ANN visualization tools, our SAM does not require target class label to find a contribution or visual explanation^25,35^.

With the proposed SAM, we investigate and compare the internal spiking behavior of two popular SNN training algorithms: surrogate gradient based training^20^ and ANN–SNN^15^ conversion on a non-trivial image dataset (*i*.*e*., Tiny-ImageNet). Then, we observe the spike representation of each layer across different time-steps to understand the temporal characteristics of SNNs. Finally, we provide a visual understanding of previously observed robustness results^36^ that SNNs are more resilient to adversarial attacks^37^. Essentially, we measure the difference of heatmaps between clean samples and adversarial samples using SAM to highlight the robustness of SNNs with respect to ANNs. Note, throughout the paper, we refer to the real-valued continuous/differentiable activation (like ReLU) based neural networks as ANNs to differentiate them from SNNs.

**Figure 2:**
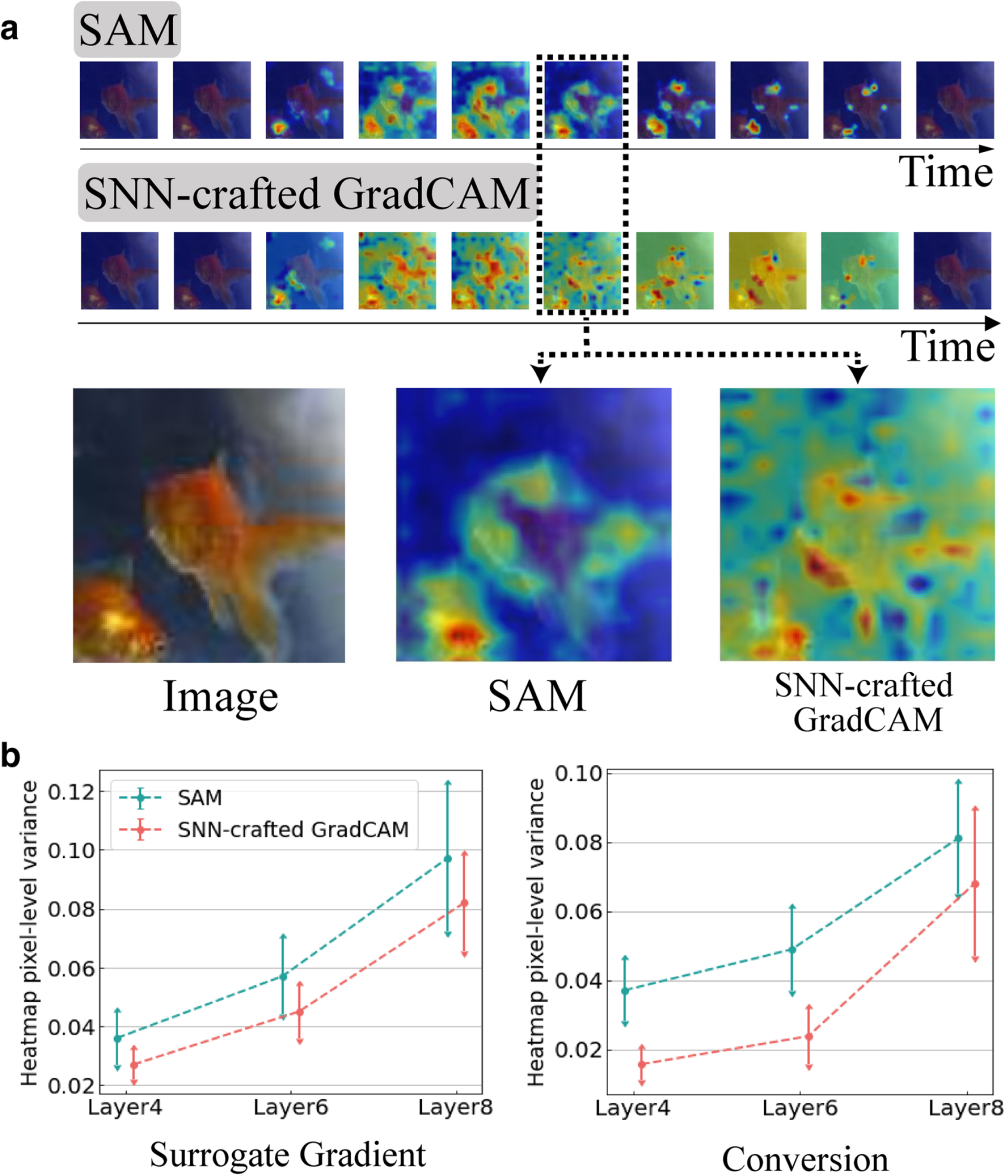
Comparison of SNN-crafted Grad-CAM and SAM. (**a**) Visualization of SNN-crafted Grad-CAM and SAM at the fourth convolutional layer in VGG11 on Tiny-ImageNet dataset. We use surrogate gradient training to train the SNN (see “Methods” for more details). The approximate backward gradient function in SNN-crafted Grad-CAM induces “heatmap smoothing effect”. In contrast, the proposed SAM visualization highlights the discriminative region of the image. Here, we normalize the heatmaps to have the value between 0 (blue) and 1 (red). We use Matplotlib (URL: https://matplotlib.org) for visualizing heatmaps shown in Figs. 2, 4, and 6. (**b**) Pixel-level variance in heatmaps obtained from SNNs trained with surrogate gradient learning and ANN–SNN conversion (see “Methods”). We report the average variance from the total samples in Tiny-ImageNet dataset. For all scenarios, SAM shows a higher heatmap variance compared to SNN-crafted Grad-CAM implying that SAM yields discriminative visualization.

## Results

### SNN-crafted Grad-CAM

Grad-CAM^25^ highlights the region of the image that highly contributes to classification results. Grad-CAM computes a backward gradient from the output classifier logits to the pre-defined target layer. After that, channel-wise contribution score is obtained by using global average pooling. Based on this, the final heatmap is defined as the weighted sum of contribution scores across all feature maps or channels. Different from conventional ANNs, SNNs take spike trains as inputs across multiple time-steps. Therefore, we can compute multiple SNN-crafted Grad-CAM heatmaps across the total number of time-steps *T*. Similar to Grad-CAM, we quantify the contribution of each channel by accumulating gradients across all time-steps:

1 $$\begin{aligned} \alpha ^{c, k} = \frac{1}{N}\sum _i\sum _j\sum _t \frac{\partial y^c}{\partial A^k_{ij, t}}. \end{aligned}$$

Here, *N* is a normalization factor, and $A^k_{ij, t}$ is the spike activation value of the *k*th channel at time-step *t*, and (i, j) is the pixel location. Note that we use a ground truth label *c* for a given image to compute the heatmap. Therefore, the channel-wise weighted sum of spike activation can be calculated as:

2 $$\begin{aligned} G^c_{ij,t} = max \left( 0, \sum _{k}\alpha ^{c,k}_{t}A^k_{ij,t} \right) . \end{aligned}$$

For a clear comparison with conventional ANN based Grad-CAM, we refer to $G^c_{ij,t}$ as “SNN-crafted Grad-CAM” in the remainder of the paper. It is worth mentioning that we convert a static image to temporal spike trains using Poisson rate coding (See “Methods” for details).

SNN-crafted Grad-CAM suffers from what we term as a “*heatmap smoothing effect*” caused by the approximated backward gradient function. To visualize the heatmap at shallow/initial layers, the gradients from the output need to pass through multiple layers using the approximated backward function (see Supplementary Note 1). The accumulated approximation error yields a non-discriminative heatmap as shown in Fig. 2a. Note that the beginning and the ending time-steps have little spike activity^20^ resulting in heatmaps with zero values. To validate the “heatmap smoothing effect” quantitatively, we compute the pixel-wise variance of the heatmap. So, the heatmap containing non-discriminative information (*i*.*e*., similar pixel values) should have lower variance. In Fig. 2b, SNN-crafted Grad-CAM shows lower variance compared to our proposed SAM (will be discussed in the next section). In SNN visualization, there are multiple heatmaps (*i*.*e*., one heatmap per time-step). So, we use the maximum variance value across all time-steps for Fig. 2b. Further, we note that the heatmap visualization in both SAM and SNN-crafted Grad-CAM in Fig. 2a varies across each time-step underlying the fact that the SNN looks at different regions of the same input over time to make a prediction. Overall, the visualization tool for SNNs requires a new perspective that can circumvent the error accumulation problem of approximate gradients or backpropagation. In all our experiments, we use VGG11^1^ architecture of SNN based on LIF neuron to perform image classification on the complex Tiny-ImageNet dataset, *i*.*e*., subset of ImageNet dataset^38^ (see Supplementary Table 1 for detailed information on the network architecture and dataset).

### Spike activation map (SAM)

SAM is a new paradigm for bio-plausible visualization of SNNs. We do not need to use any class label or perform backpropagation to calculate gradients. SAM only uses the spike activity in forward propagation to calculate heatmaps. Thus, this visualization is not just for a specific class but highlights the regions that the network focuses on for any given image. Surprisingly, we observe that SAM shows meaningful visualization even without any ground truth labels (Fig. 2a). Mathematically, our objective can be formulated as finding a mapping function $f(\cdot )$:

3 $$\begin{aligned} M_{t} \leftarrow f(S_{0}, S_{1}, \ldots , S_{t-1}), \end{aligned}$$

where, $M_{t}$ is SAM and $S_{t}$ is spike activity at time-step *t*. We leverage the biological observation that spikes with short inter-spike interval (ISI) highly contribute to the neural decision process^32–34^. This is because short ISI spikes are more likely to stimulate post-synaptic neurons, conveying more information^33,39,40^. To apply this to our visualization method, we first define the temporal spike contribution score (TSCS). For a given neuron, TSCS evaluates the contribution of a previous spike at time $t'$ with respect to current time *t*. It is natural that the contribution of the previous spike with respect to the current neuronal state will decrease as time progresses. Therefore, the TSCS value can be formulated as:

4 $$\begin{aligned} T(t, t') = \exp (- \gamma |t-t'|), \end{aligned}$$

where, $\gamma$ is a hyperparameter which controls the steepness of the exponential kernel function.

**Figure 3:**
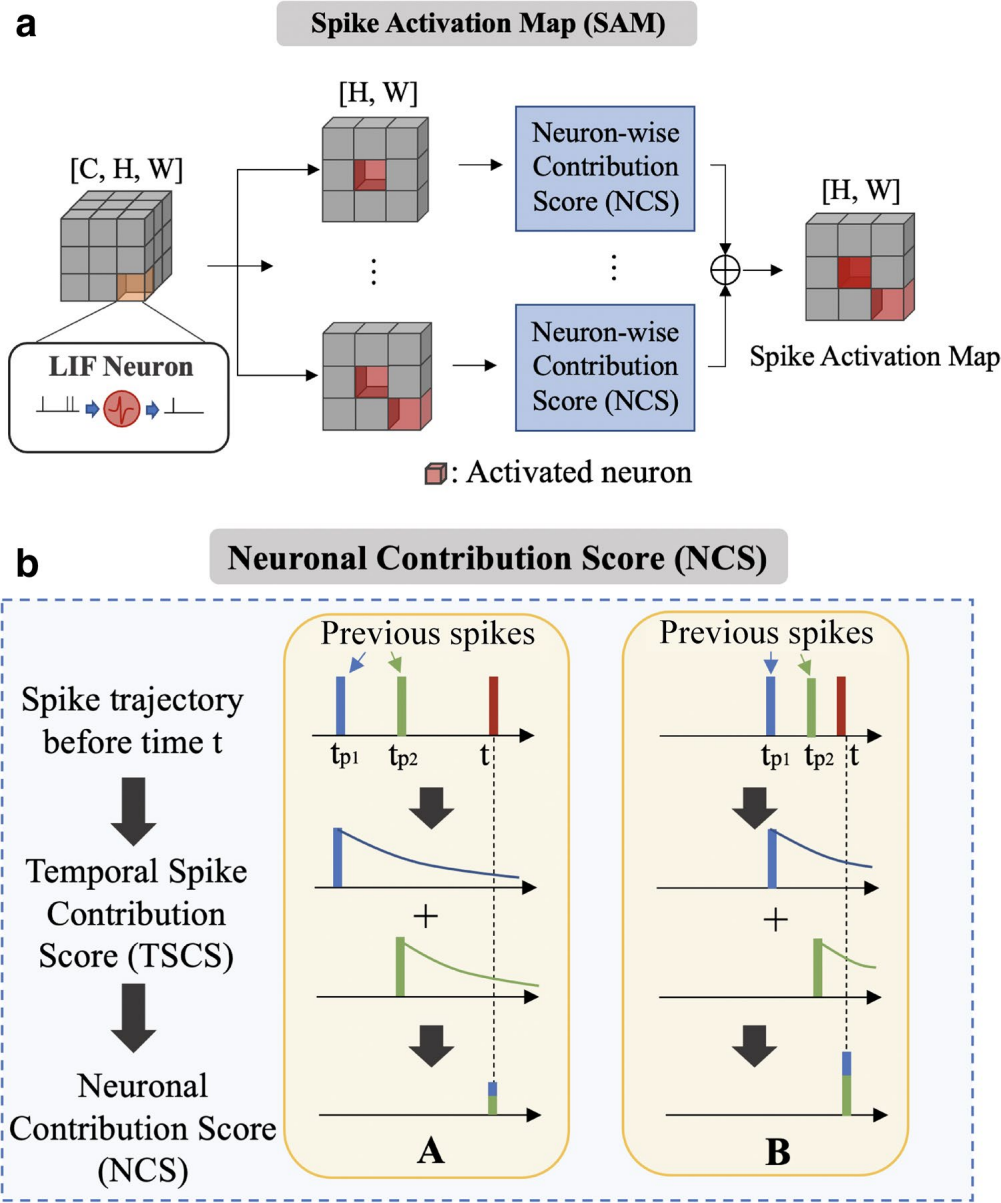
Overall process of SAM. (**a**) Illustration of spike activation map (SAM). We illustrate an intermediate feature tensor with channel *C*, height *H*, and width *W*. For each channel, we compute a neuron-wise contribution score. After that, we sum all neuronal contribution score (NCS) along the channel axis to obtain the SAM heatmap. (**b**) The NCS for each neuron is based on the previous spike trajectory. For every spike, we define temporal spike contribution score (TSCS) with an exponential kernel. We take into account TSCS from previous spikes in order to compute NCS. Thus, NCS shows high value when more spikes exist in a short time window.

**Figure 4:**
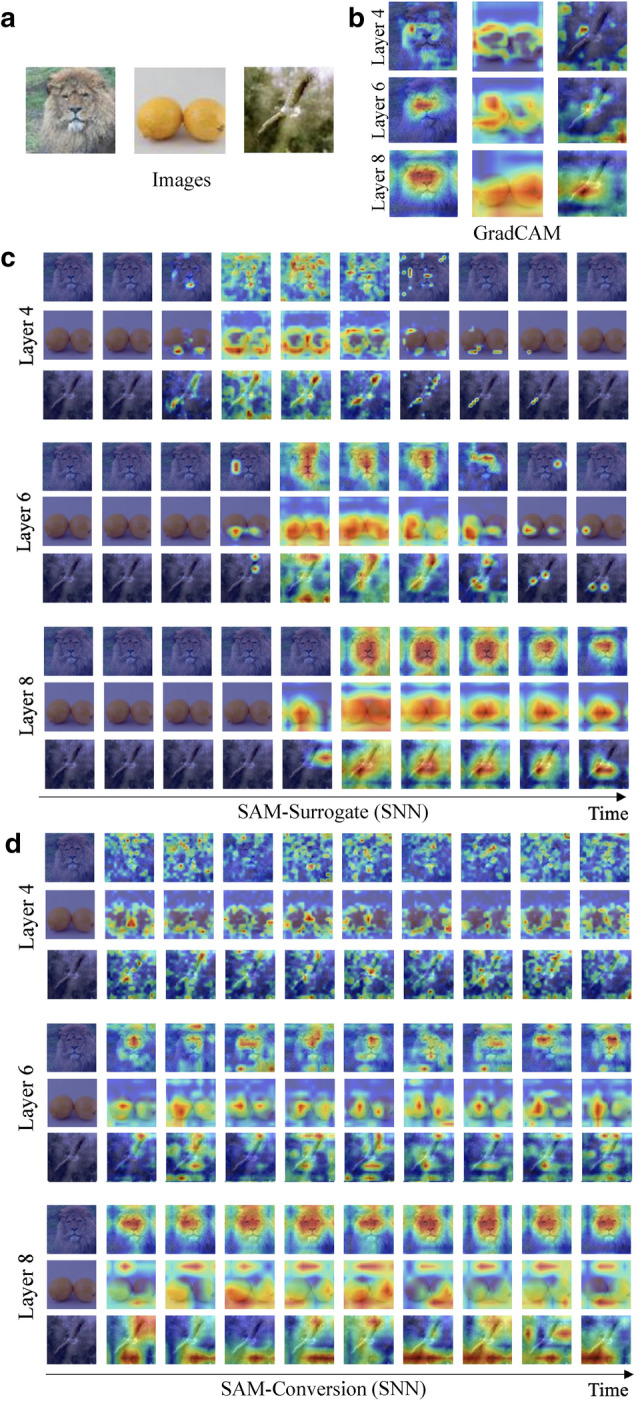
Visualization of SAM. (**a**) Original images. (**b**) Grad-CAM results on reference ANN. (**c**) SAM results on SNN trained with surrogate gradient. (**d**) SAM results on SNN trained with ANN–SNN conversion. We visualize the internal spike representation of VGG11 using SAM at layer 4, layer 6, and layer 8. We show the visualization for 10 uniformly sampled time-steps. It is worth mentioning Grad-CAM exploits ground truth labels but our SAM can be obtained without any label information. Here, the networks are trained on Tiny-ImageNet dataset. We provide more visualization results in Supplementary Figs. 2–7.

**Figure 5:**
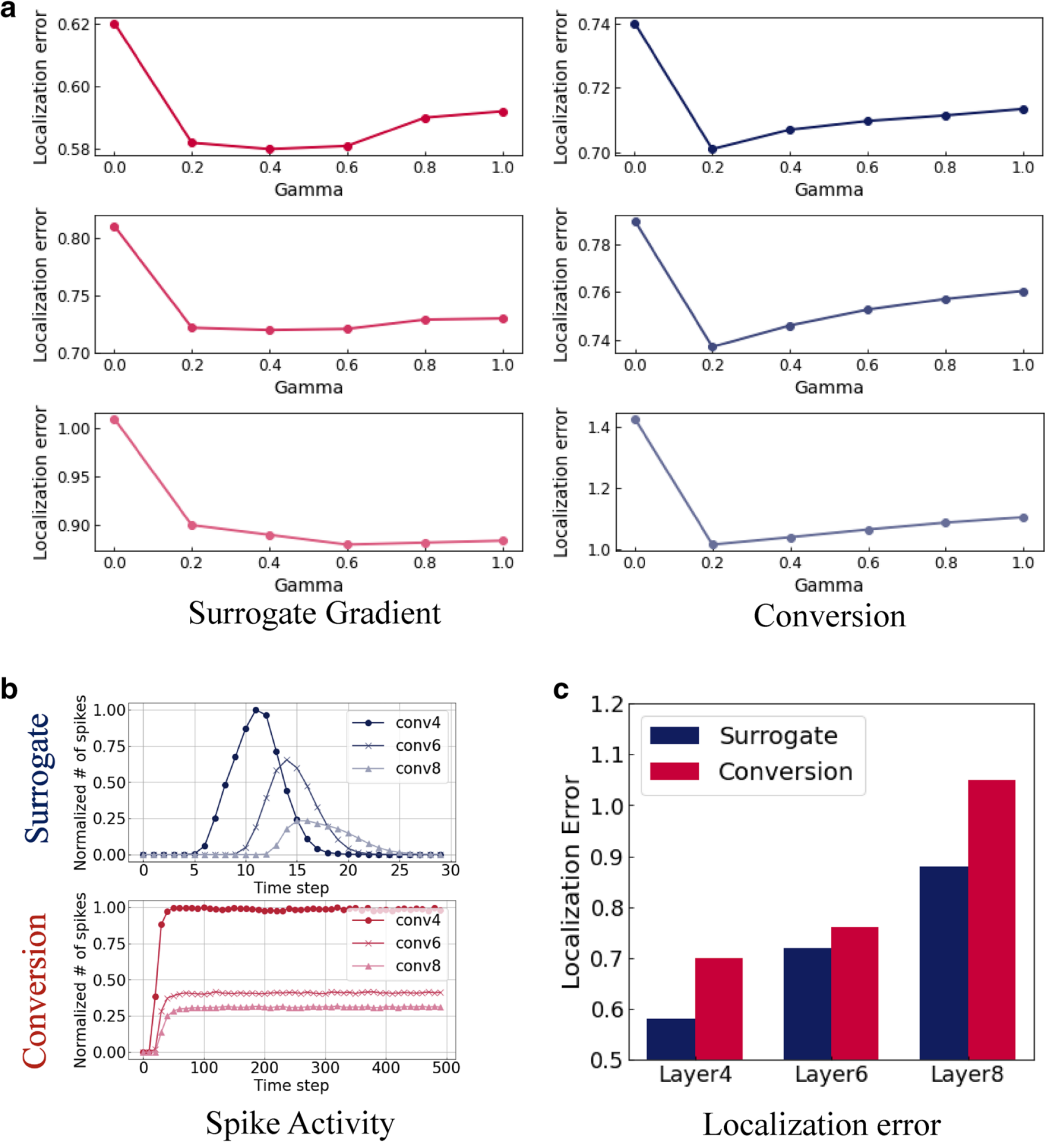
Localization error of SAM. We measure the localization error (*i*.*e*., the difference between SAM and Grad-CAM) in various SNN training configurations (See *Methods*). For all experiments, we train a VGG11 network on Tiny-ImageNet dataset. (**a**) Localization error at layer 4 (top row), layer 6 (middle row), and layer 8 (bottom row) with respect to hyperparameter $\gamma$. The results show that zero $\gamma$ value does not consider temporally evolving characteristic of SNNs and results in highest localization error. (**b**) Illustration of the normalized number of spikes with time. The spike activity with surrogate training shows Gaussian-like trend. On the other hand, a conversion approach yields nearly constant values after timestep 50. The characteristic of activity is related to visualization results in Fig. 4. (**c**) Localization error comparison across different layers. The localization error increases with deeper layers since the visualization tool focuses on more selective information in deep layers. For all layers, the conversion method shows a higher localization error compared to the surrogate learning method.

**Figure 6:**
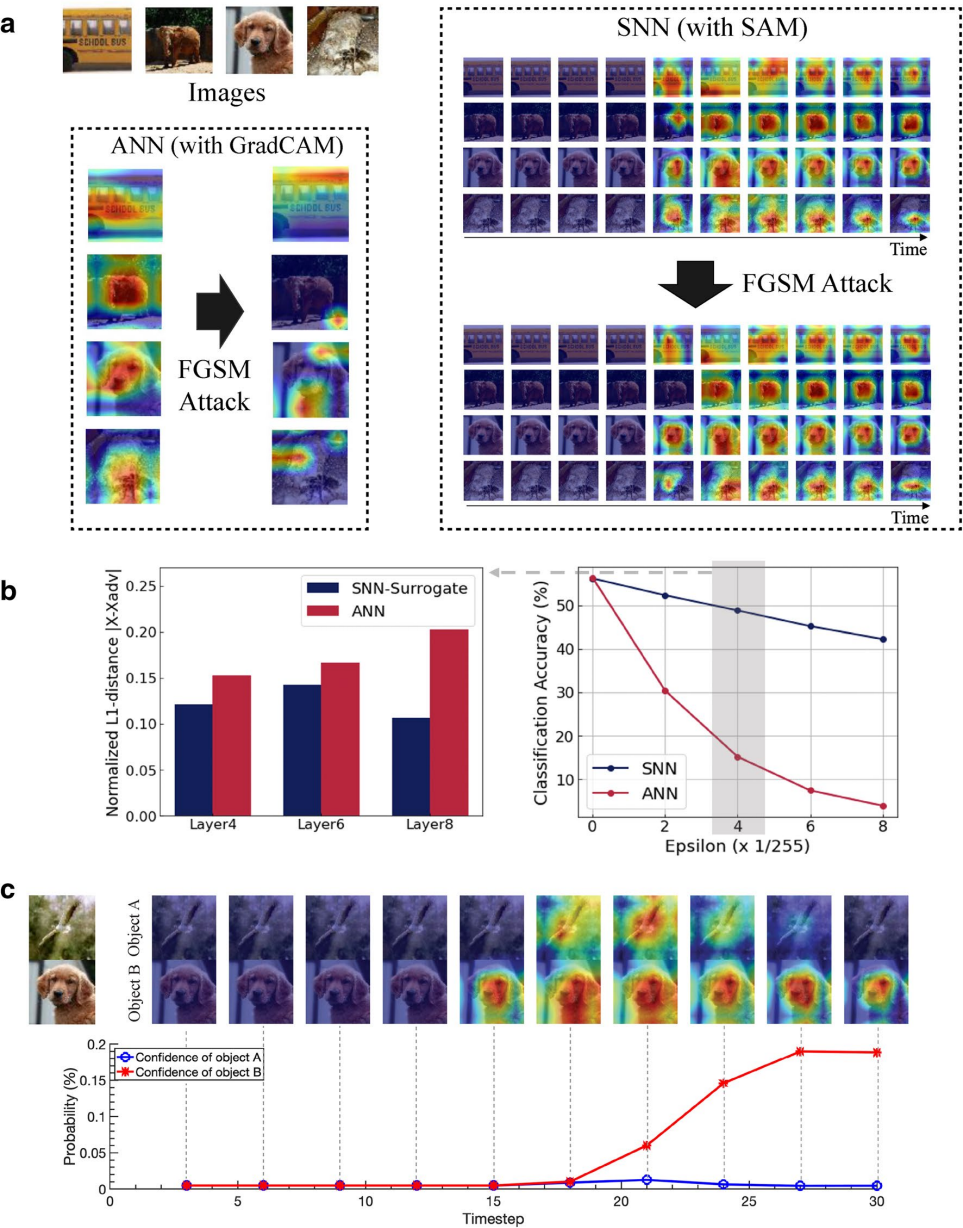
Visualizing robustness and sensory suppression behavior of SNN with SAM. We use the VGG11 networks with Tiny-ImageNet dataset. (**a**) Visualization of robustness with SAM. We show the Grad-CAM and SAM results with respect to the clean and adversarial images. Heatmap from SNN with SAM shows less variation compared to the ANN counterpart (see Supplementary Fig. 8 for additional visualization results). (**b**) Classification accuracy with respect to varying attack strengths of Fast Gradient Sign Method (FGSM) attack. We compute the normalized L1 distance between heatmaps for clean *X* and adversarial inputs $X_{Adv}$ at $\epsilon = \frac{4}{255}$. For SNN, we report the maximum L1 distance across multiple time-steps. (**c**) Visualization of SAM for multi-object images. We concatenate two images vertically and visualize the region where the networks focuses on. Note, since we use Global Average Pooling after the convolutional feature extractor, the networks can make predictions regardless of the input image resolution. The network attends one of the two objects at the end of the time-steps. We also provide the probabilities of two classes predicted from the output classifier of the VGG11 model across time.

To consider multiple previous spikes, we define a set $P_{ij}^k$ that consists of previous firing times of a neuron at location (*i*, *j*) in *k*th channel. For every time-step, we compute a neuronal contribution score (NCS) $N^k_{ij,t}$ at time-step *t*, by summing all TSCS values of previous spikes in $P_{ij}^k$:

5 $$\begin{aligned} N^k_{ij,t} = \sum _{t' \in P_{ij}^k} T(t, t'). \end{aligned}$$

Thus, a neuron has high NCS if it spikes frequently over a short time interval and vice-versa. Finally, we calculate the SAM heatmap $M_{ij,t}$ at time-step *t* and location (*i*, *j*) by multiplying spike activity $S_{ij,t}$ with NCS value $N_{ij,t}$ and summing across all *k* channels:

6 $$\begin{aligned} M_{ij,t} = \sum _{k} N_{ij,t}^k S^k_{ij,t}. \end{aligned}$$

We illustrate the overall flow of SAM in Fig. 3a. For every neuron, we compute NCS and add the values across the channel axis in order to get SAM. In Fig. 3b, we depict two examples (case *A* and case *B*) for calculating NCS. In case *A*, the previous spikes occur at time-step $t_{p1}$ and $t_{p2}$ that are reasonably earlier than the current spike time *t*. As a result, the contribution of previous spikes is small due to the exponential kernel. On the other hand, in case *B*, $t_{p1}$ and $t_{p2}$ are close to the current spike time *t*. In this case, the neuron has a high NCS value.

In Fig. 4, we visualize the qualitative results of SAM on SNNs trained with surrogate learning (Fig. 4c) as well as ANN–SNN conversion (Fig. 4d). We also show the Grad-CAM visualization obtained from a corresponding ANN for reference (Fig. 4b). Note that SAM does not require any class label, while Grad-CAM uses ground truth labels to create heatmaps. Interestingly, heatmaps obtained from SAM across different time-steps on SNNs shows a similar result with Grad-CAM on ANNs. The region of interest in SAM is highlighted in a discriminative fashion. This supports our assertion that SAM is an effective visualization tool for SNNs. Moreover, the results imply that ISI and temporal dynamics can yield intepretability for deep SNNs. So far, no studies have analysed the underlying information learnt in different layers of an SNN. It has been always assumed that SNNs like ANNs learn features in a generic-to-specific manner as we go deeper. For the first time, we visualize the explanations at intermediate layers of SNNs to support this assumption. Interestingly, with surrogate learning, the SAM visualization (Fig. 4c) shows that shallow layers of SNNs represent low-level structure and deep layers focus on semantic information. For example, layer 4 highlights the edges or blobs of the lion, such as eyes and nose. On the other hand, layer 8 highlights the full face of the lion. More visualization results are provided in Supplementary Figs. 2–7.

Further, we conduct ablation studies to understand the effect of hyperparameter $\gamma$ on SAM in Eq. 4. The $\gamma$ value decides the steepness of the exponential kernel function in TSCS. A kernel with high $\gamma$ takes into account very recent spike history, where as low $\gamma$ considers longer spike history. In Fig. 5a, we visualize the localization error with respect to $\gamma$ for different layers in VGG11 SNN for conversion and surrogate gradient training methods. For both methods, $\gamma = 0$ shows the highest localization error since the kernel does not filter redundant and irrelevant long ISI spikes. Another interesting observation is that the localization error increases for large gamma value (*e*.*g*., 1.0). This is because high $\gamma$ limits reliable visualization considering only very recent spikes and ignores spike history to a large extent.

### Comparison between surrogate gradient learning and conversion

We compare the SAM visualization results of surrogate gradient learning and ANN-SNN conversion in Fig. 4c, d. From the figure, we observe a trend in the heatmap visualization of surrogate gradient learning with zero activity at early time-steps leading to discriminative activity in the mid-range followed by zero activity again towards the end. In contrast, conversion maintains similar heatmaps during the entire time period. This is related to the variation in spike activity for each time-step as shown in Fig. 5b. Since surrogate gradient learning considers temporal dynamics during training^6,20^, each layer passes the information (*i*.*e*., the number of spikes) continuously over time. On the other hand, conversion does not show any temporal propagation (see Supplementary Fig. 1 for more detailed explanation). Moreover, we observe that surrogate gradient learning has more accurate (*i*.*e*., similar to Grad-CAM from ANN) heatmaps highlighting the region of interest across all layers. Notably, the conversion method highlights only partial regions of the object (*e*.*g*., lemon) and in some cases (*e*.*g*., bird) the wrong region. This observation is supported by the localization error comparison in Fig. 5c. For all layers, surrogate gradient learning shows lower localization error. It is evident that conversion methods do not account for any temporal dynamics during training^6^. We believe that this missing temporal dependence accounts for less interpretability. Thus, we assert that SNNs obtained with surrogate gradient learning (incorporating temporal dynamics) are more interpretable. Therefore, all visualization analyses in the following subsections focus on the surrogate gradient learning method.

### Adversarial robustness of SNN

Different from a human visual system, neural networks are vulnerable to *adversarial attacks*. These attacks are created by adding small, yet, structured perturbations to the input image^37^. Previous studies^36,41^ have asserted that SNNs trained with surrogate gradients are more robust to adversarial inputs than ANNs. In order to show the effectiveness of SNNs under adversarial noise attack, we conduct a qualitative and quantitative comparison between Grad-CAM and SAM. We attack both ANN and SNN using Fast Gradient Sign Method (FGSM) attack^37^ and SNN-crafted FGSM attack^36^ with $\epsilon = \frac{4}{255}$ (see “Methods” and Supplementary Note 3 for implementation details). In Fig. 6a, we observe that Grad-CAM shows large change in visualization before/after attacking the ANN. In fact, the ANN after attack starts focusing on random parts of the image and therefore misclassifies the adversarial inputs. On the other hand, SAM shows almost similar results before/after attack. Interestingly, we observe that SAM in case of adversarial attack slightly changes at the earlier time-steps with respect to the clean input visualization. But, as time progresses, the visualization between the adversarial input and the clean input look similar highlighting suitable regions of interest. This implies that the temporal processing in SNNs enables compensation and correction of any noise in the input. We surmise that accumulating temporal information in SNNs imparts robustness to the system. Further, we show the classification accuracy with respect to the attack intensity, and normalized L1-distance between heatmaps of clean and adversarial images at $\epsilon = \frac{4}{255}$ in Fig. 6b. The results show that SNN is more robust than ANN in terms of both accuracy and visualization (see Supplementary Fig. 9 for additional experiments).

Surrogate learning will be more interpretable since it inherits better temporal dynamics is a widely-adopted intuition. Similarly, it is a widely accepted notion that temporal SNNs are more resilient to adversarial attacks than ANNs. However, with SAM, for the first time, we are able prove and explain our intuitions using visual explanations. Thus, SAM is a gateway to *interpretable neuromorphic computing*. For instance, SAM can enable SNN deployment for secure and intelligent systems (*e*.*g*., military defense) where robustness and interpretability (to gain user’s trust in the prediction made by the model) are paramount.

### Sensory suppression behavior of SNN

Neuroscience studies have suggested that human brain undergoes^42–44^ “sensory suppression”. That is, the brain focuses on one of multiple objects when these objects are presented at the same time. Co-incidentally, with SAM, we observe that SNNs also emulate sensory suppression when presented with multiple objects. To show this, we concatenate two randomly chosen images from Tiny-ImageNet dataset and pass the concatenated image into the SNN trained with surrogate gradient learning. Interestingly, as shown in Fig. 6c, neurons compete in the earlier time-steps for attending to both objects and finally focus/attend on only one object at later time-steps. Note, for each image, the final prediction from the SNN matches the final heatmap shown by SAM. For each timestep, we also visualize the confidences of two classes in the last layer (*i*.*e*., classifier). The confidence of each object is also varying according to the attended area by the network. These results unleash the bio-plausible characteristics of SNNs and further establish SAM as a suitable interpretation tool (Supplementary Fig. 10 provides more examples).

## Discussion

We propose a visualization tool for SNNs, called SAM. For the first time, we show the interpretability-related advantages of temporal SNNs over static ANNs. We leverage the temporal dynamics of SNNs to compute a neuronal contribution score in forward propagation based on the history of previous spikes. This is different from a conventional ANN visualization tool since SAM does not require any target labels and backpropagated gradients. Without any label, SAM highlight the discriminative region for prediction. We also compare two representative training methods in SNNs: ANN–SNN Conversion and Surrogate Gradient Backpropagation. ANN–SNN conversion method^15–18^ converts a pre-trained ANN to an SNN. Since networks are trained in the ANN domain, the training complexity is significantly removed. With careful threshold (or weight) balancing^17^, ANN–SNN conversion shows good performance on large-scale datasets. It is worth mentioning that temporal dynamics are not considered in the process of training for converted SNNs. Recently, training SNNs with spike-based backpropagation^20,45–47^ has received a lot of attention because it accounts for temporal neuronal dynamics with surrogate gradients. Our results show that surrogate methods which have explicit temporal dependence during training are more interpretable than conversion.

The interpretation of prediction in artificial neural networks has received considerable attention due to its practicality in real-world scenarios. Class Activation Map (CAM)^35^ highlights the discriminative region of an image by using a global average pooling layer at the end of the feature extractor. The CAM heat map is obtained by summing the feature maps at the last convolutional layer. Several variations of CAM have been proposed^48–50^. However, the necessity of the global average pooling layer in CAM limits its usage. To address this issue, Selvaraju et al. proposed Grad-CAM^25^, which is the generalized version of CAM. Grad-CAM computes backward gradients from the classifier to a given intermediate layer where visual explanation is required. Thus, the contribution of each neuron to the classification result can be quantified with the corresponding gradient value. Then, a 2D heatmap is obtained by using the weighted sum of the activations across the channel axis based on the gradient value. In this work, we justify that directly applying Grad-CAM to calculate visual explanations in SNNs does not yield accurate results. This is due to the non-differentiable nature of LIF neuron that interferes with Grad-CAM as well as the non-dependence of Grad-CAM on temporal dynamics.

In the neuromorphic engineering domain, there are several works that use the neuronal activity or weight connection as a visualization tool. The authors of^51^ propose a real-time graph visualization tool for analyzing connectivity and biophysical processes (with no regard to interpreting a model’s decision). Our work also has the same objective in terms of revealing internal spike behavior. However, SAM aims to visualize the region-of-interest in static images to understand the prediction made by the SNN. Our visualization is on similar lines as the visual explanation works in ANN counterparts such as, CAM and Grad-CAM. Demin and Nekhaev^52^ visualize the receptive field of neurons in 2-layers SNNs. For the output layer, they use the correlation between forwarding and reciprocal weight matrix in order to compute the receptive fields. For the hidden layer neurons, weight connection to the input layer can be directly used as a pixel-level score for receptive fields. However, their method is limited to two-layer networks with reciprocal connection. Therefore, it is difficult to apply their approach to deep SNNs for complex dataset interpretation. Deng et al.^53^ visualize the accumulated spikes in the input layer by converting input spike streams to frame-based representation. Specifically, they accumulate spikes from a Dynamic Vision Sensor (DVS) camera whenever movement happens to understand the characteristics of the DVS image. However, there is no relation to interpretation of the model prediction (e.g., where does the shallow/deep SNN layers focus to make a prediction?).

Our bio-inspired SAM presents a promising new technique for building robust and hardware-friendly visual reasoning systems. Specifically, in this work, we observed that SNNs with SAM provide robust explanation results with respect to adversarial noise over ANN counterparts. A robust interpretation tool is essential for deploying intelligent systems in ubiquitous scenarios (such as, self-driving cars, health care monitoring systems, defense etc.). A huge advantage of the proposed SAM is that it is hardware friendly since all computations to calculate the visual explanation are in forward propagation. Inference-only hardware accelerators do not comprise of backpropagation and gradient calculation modules since they only perform forward propagation calculations. Thus, SAM can be easily integrated into state-of-the-art accelerators and neuromorphic computing engines^12–14^. SAM requires memory to store the ISI and a simple computation module (*e*.*g*., Look-Up-Table) for implementing the exponential kernel, which can be easily implemented in an inference accelerator with marginal cost overhead. On the other hand, GradCAM, a widely used visualization tool for conventional ANN, requires a backpropagation module and a huge memory to store the computational graph for gradients. Therefore, our SAM paves the path towards practical and interpretable neuromorphic computing. In this work, we use surrogate gradient learning based (with rate coding) and ANN–SNN conversion methods. This is because these optimization algorithms allow training on large-scale datasets and deeper convolutional architectures. Another prospective way of training SNNs is spike-timing-based learning algorithms^54–56^, where each neuron fires only once across all time-steps. Thus, spike-timing based learning requires a smaller number of spikes compared to the other algorithms. However, their efficacy is still limited to small-scale datasets (i.e., MNIST) and shallow architectures, where it can be difficult to show meaningful information with SAM. In the future, as the development of spike-timing-based learning algorithms enables more complex datasets and architecture, it will be intriguing to study and analyze SAM with them.

## Methods

### Leaky-integrate-and-fire neuron

Leaky-Integrate-and-Fire (LIF) neuron is the main component of SNNs. The internal state of an LIF neuron is represented by a membrane potential $U_m$. As time progresses, the membrane potential decays with time constant $\tau _m$. Given an input signal *I*(*t*) and an input resistance *R* at time *t*, the differential equation of the LIF neuron can be formulated as:

7 $$\begin{aligned} \tau _m \frac{dU_m}{dt} = -U_m + RI(t). \end{aligned}$$

This continuous dynamic equation is converted into a discrete equation for digital simulation. More concretely, we formulate the membrane potential $u_{i}^{t}$ of a single neuron *i* as:

8 $$\begin{aligned} u_i^t = \lambda u_i^{t-1} + \sum _j w_{ij}o^t_j - \theta o^{t-1}_i, \end{aligned}$$

where, $\lambda$ is the leak factor, $w_{ij}$ is the weight of the connection between pre-synaptic neuron *j* and post-synaptic neuron *i*. If the membrane potential $u_i^{t-1}$ exceeds a firing threshold $\theta$, the neuron *i* generate spikes $o_i^{t-1}$, which can be formulated as:

9 $$\begin{aligned} o^{t-1}_i = {\left\{ \begin{array}{ll} 1, &{} \text {if } u_i^{t-1}>\theta , \\ 0 &{} \text {otherwise.} \end{array}\right. } \end{aligned}$$

After the neuron fires, we perform a soft reset, where the membrane potential value is lowered by threshold $\theta$. Because of this non-differentiable firing behavior, training SNNs with gradient learning is a huge challenge^6^. To address this issue, previous studies^45,46^ approximate the backward gradient function (*e*.*g*., piecewise linear and exponential) to implement gradient learning.

### Poisson rate coding

To convert a static image into multiple binary spikes, we use Poisson rate coding, or rate-based coding. This shows outstanding performance among other spike coding schemes such as temporal^56^, phase^57^, and burst^58^. Poisson coding generates a spike train over multiple time-steps where the number of spikes is approximately proportional to the pixel intensity of the input image. In practice, we compare each pixel value with a random number [0, 255] at every time-step. If the generated random number is less than the pixel intensity, the Poisson spike generator does not produce spikes, otherwise, it generates a spike with amplitude 1. The generated spikes are then passed through an SNN.

### Surrogate gradient backpropagation

In this paper, we visualize the internal spike behavior of two representative and widely-used training methods: surrogate gradient training^20^ and ANN–SNN conversion^15^. Since ANNs can be trained with well-established optimization methods and frameworks, SNNs from ANN–SNN conversion shows reliable performance on very large-scale datasets (*e*.*g*., ImageNet). In contrast, most surrogate gradient training methods are limited to small datasets (*e*.*g*., MNIST and CIFAR10) due to approximated backward gradients^7,20,45,47^. These simple datasets are too small to be analyzed by visualizing heatmap. But, the authors in^20^ recently proposed a temporal batch normalization technique, called Batch Normalization Through Time (BNTT), for surrogate gradient learning of SNNs on large-scale datasets. We use this algorithm for all our surrogate gradient training experiments.

The SNN-crafted batch normalization layer^20^, called BNTT, improves training stability and reduces latency on classification tasks while preserving accuracy. We add the BNTT layer before an LIF neuron. Therefore, the weighted pre-synaptic input spikes are normalized as:

10 $$\begin{aligned} u_i^t = \lambda u_i^{t-1} + \gamma _i^t \left( \frac{\sum _j w_{ij}o^t_j - \mu ^t_i}{\sqrt{(\sigma _i^t)^2 + \epsilon }} \right) - \theta o^{t-1}_i, \end{aligned}$$

where, $\gamma _i^t$ is a learnable parameter in the BNTT layer, $\epsilon$ is a small constant for numerical stability, the mean $\mu _{i}^t$ and variance $\sigma _{i}^t$ are calculated from the samples in a mini-batch for each time step *t*. We append all intermediate layers of an SNN with a BNTT layer. At the output layer, we set the number of output neurons to the number of classes *C*. At the output, we accumulate the spikes over all time-steps by fixing the leak parameter $\lambda$ (Eq. 8) as one to prevent information loss from leakage. This stacked voltage is converted into a probability distribution using a softmax layer. Finally, we compute the cross-entropy loss as:

11 $$\begin{aligned} {L} = - \sum _{i} y_{i} log \left( \frac{e^{u_i^T}}{\sum _{k=1}^{C}e^{u_k^T}} \right) . \end{aligned}$$

Here, $y_i$ represents the ground truth label, and *T* is the total number of time-steps. Then, we accumulate the backward gradients over all time-steps (see Supplementary Note 1 for details on BNTT surrogate learning).

### ANN–SNN conversion

We use the threshold normalization method proposed in^15^ for implementing the ANN–SNN conversion method. The firing threshold ($\theta$ in Eq. 9) is normalized with respect to real spike inputs to account for actual SNN operation in the conversion process. First, we copy the weight parameters of a pre-trained ANN to an SNN. Then, for every layer, we compute the maximum activation across all time-steps and set the firing threshold to the maximum activation value. The conversion process sets the threshold in a layerwise manner, starting from the first layer and sequentially going through deeper layers (See Supplementary Note 2 for more details). Note that we do not use batch normalization in conversion^59^ since all input spikes have zero mean values. Also, following the previous works^15–17^, we use Dropout^60^ for both ANNs and SNNs during conversion.

### Fast gradient sign method (FGSM) attack

Previous studies show that deep neural networks are vulnerable to adversarial inputs. Adversarial patch methods add a small patch on an image. This patch induces adversarial effect on the networks. However, Subramanya et al.^61^ assert that these methods are easily detected by Grad-CAM, which limits its practicality. Another way to generate adversarial attack is adding imperceptible noise to an input image. FGSM^37^ is a widely-used and fundamental attack technique. FGSM computes the sign of the gradient in the direction of reducing the confidence of the original prediction. Recently, Sharmin et al.^36^ proposed an SNN-crafted FGSM attack. They accumulate gradients across all time-steps. More detailed explanation on FGSM attack is provided in Supplementary Note 3.

### Dataset and network

To conduct comprehensive analysis, we carefully select the dataset for our experiments. This is because smaller datasets such as MNIST^62^, CIFAR10 and CIFAR100^63^ have too low resolution (*e*.*g*., $28 \times 28$ or $32 \times 32$) to yield any meaningful visualization. ImageNet dataset has a high image resolution but directly training SNNs with surrogate gradient becomes hard and time-taking. Therefore, we conduct a case study on the Tiny-ImageNet which is a subset of the original ImageNet dataset. Tiny-ImageNet consists of 200 different classes of ImageNet dataset^38^, with 100,000 training and 10,000 validation images. The resolution of the images is $64 \times 64$ pixels. Our implementation is based on Pytorch^64^. We adopt a VGG11 architecture for both ANNs and SNNs (see Supplementary Table 1) . For ANN–SNN conversion, we use 500 time-steps with firing threshold scaling^16^. For surrogate gradient BNTT training, we train the networks with standard SGD with momentum 0.9, weight decay 0.0005, time-steps 30. The base learning rate is set to 0.1. We use step-wise learning rate scheduling with a decay factor 10 at [0.5, 0.7, 0.9] of the total number of epochs. We set the total number of epochs to 90. We set the leak factor of SNN with surrogate gradient learning and conversion to 0.99 and 1, respectively. For visualization, we uniformly sample 10 images for both surrogate gradient learning and conversion.

### Evaluation metric for localization error

To quantitatively compare the SAM visualization of conversion and surrogate gradient methods, we define a metric called localization error. Localization error of SAM visualization is calculated using Grad-CAM visualization (obtained from ANNs) as a reference. To quantify the error between SAM and Grad-CAM, we compute the cross entropy function between the predicted SAMs $M_{t}$ (one SAM for one time-step) and a Grad-CAM *G* from ANN. Then we select the minimum error across all time-steps and define the minimum value as localization error.

12 $$\begin{aligned} E = \min _{t} \left\{ \frac{1}{N} \sum _{i,j} G_{ij}\log (M_{ij,t}) + (1-G_{ij})\log (1-M_{ij,t}) \right\} . \end{aligned}$$

Here, *N* is normalization factor and (i, j) indicates a pixel location.

## Supplementary Information

Supplementary Information 1.

Supplementary Information 2.

Supplementary Information 3.

Supplementary Information 4.
