# Supplementary figures and images for: Visual explanations from spiking neural networks using inter-spike intervals
Source: Sci Rep. 2021 Sep 24;11:19037. doi: 10.1038/s41598-021-98448-0 (PMC8463578; doi:10.1038/s41598-021-98448-0)

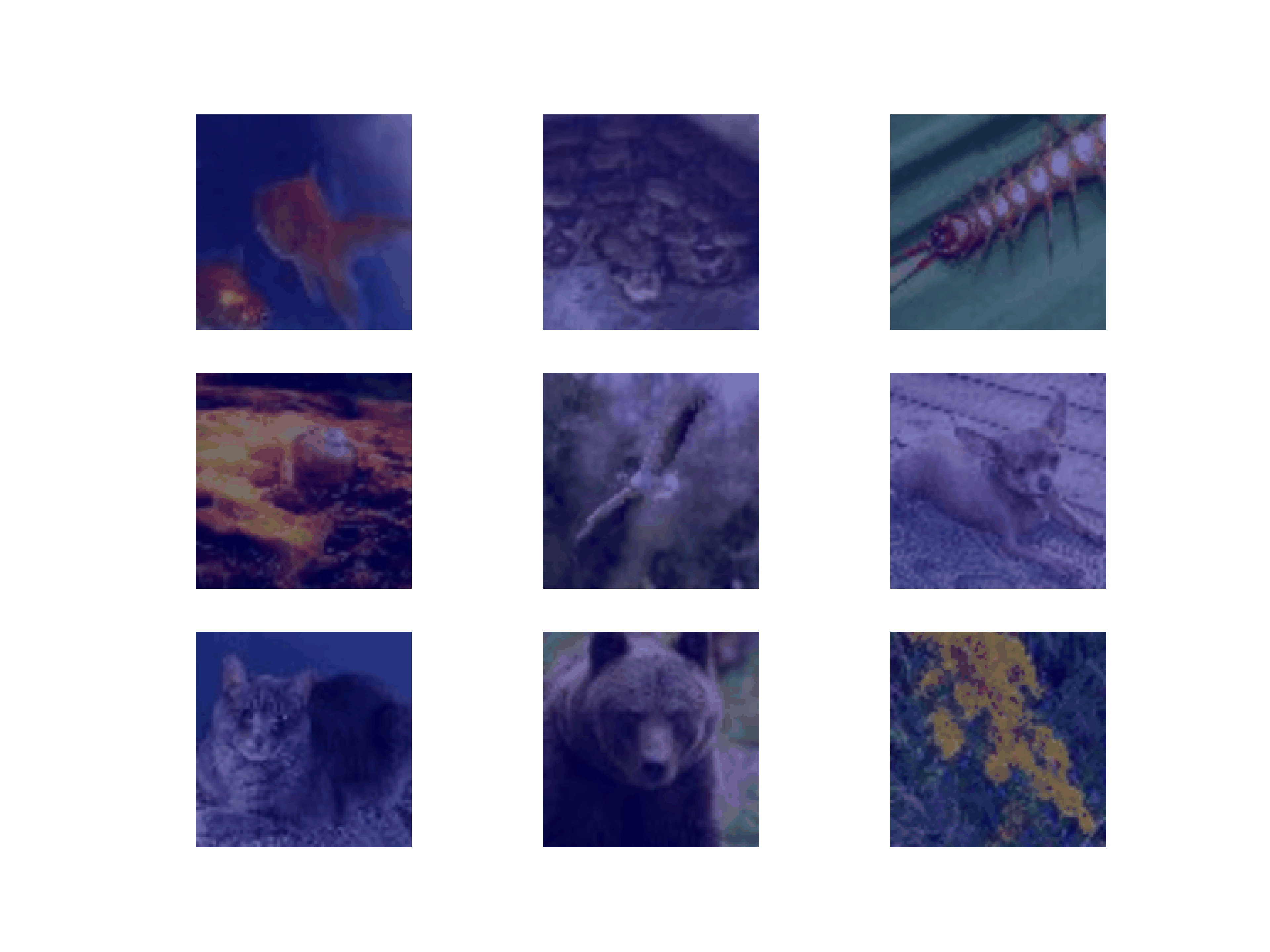

Supplement: Supplementary file 2 — Supplementary Information 2. [file 41598_2021_98448_MOESM2_ESM.gif]

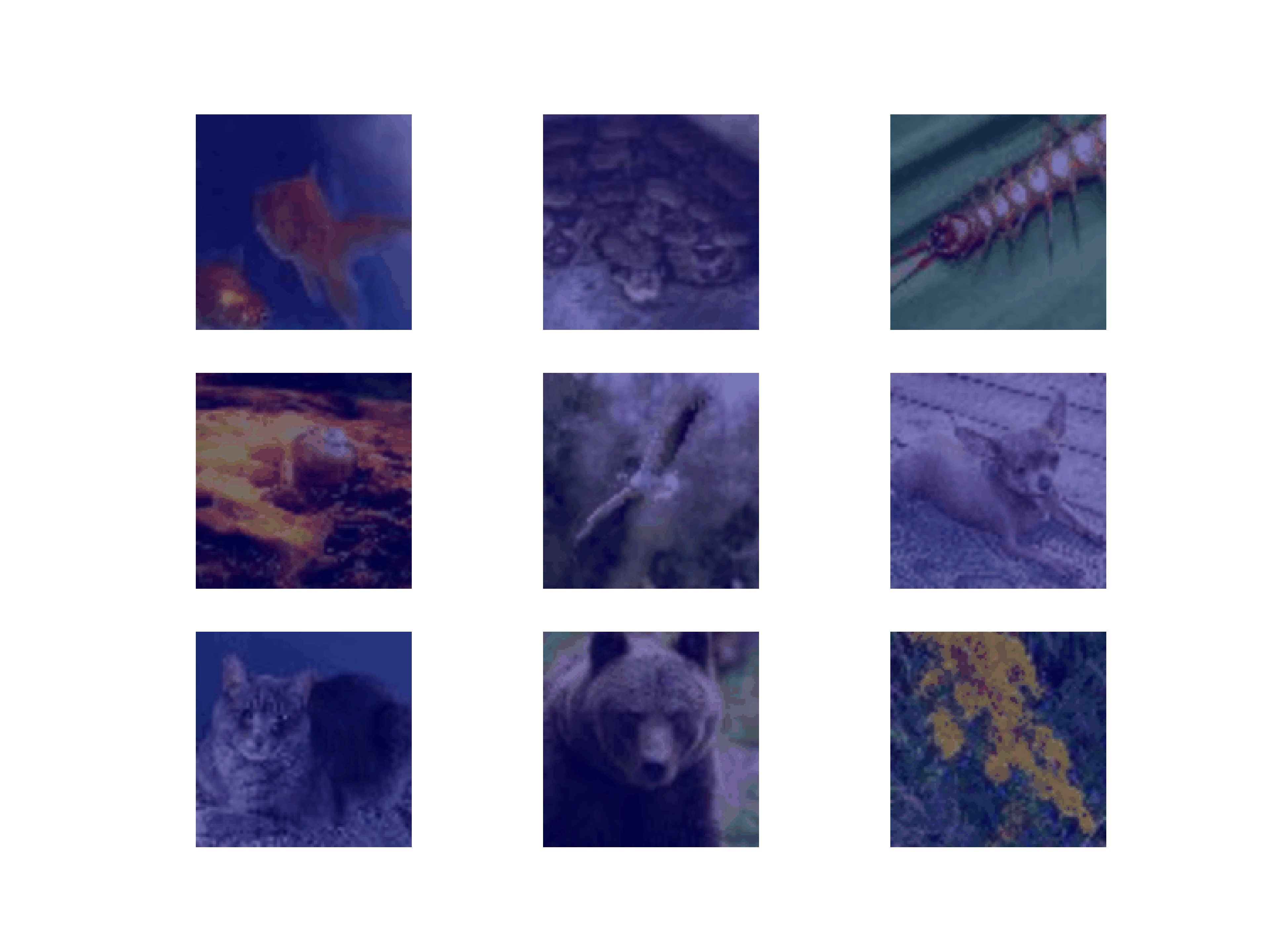

Supplement: Supplementary file 3 — Supplementary Information 3. [file 41598_2021_98448_MOESM3_ESM.gif]

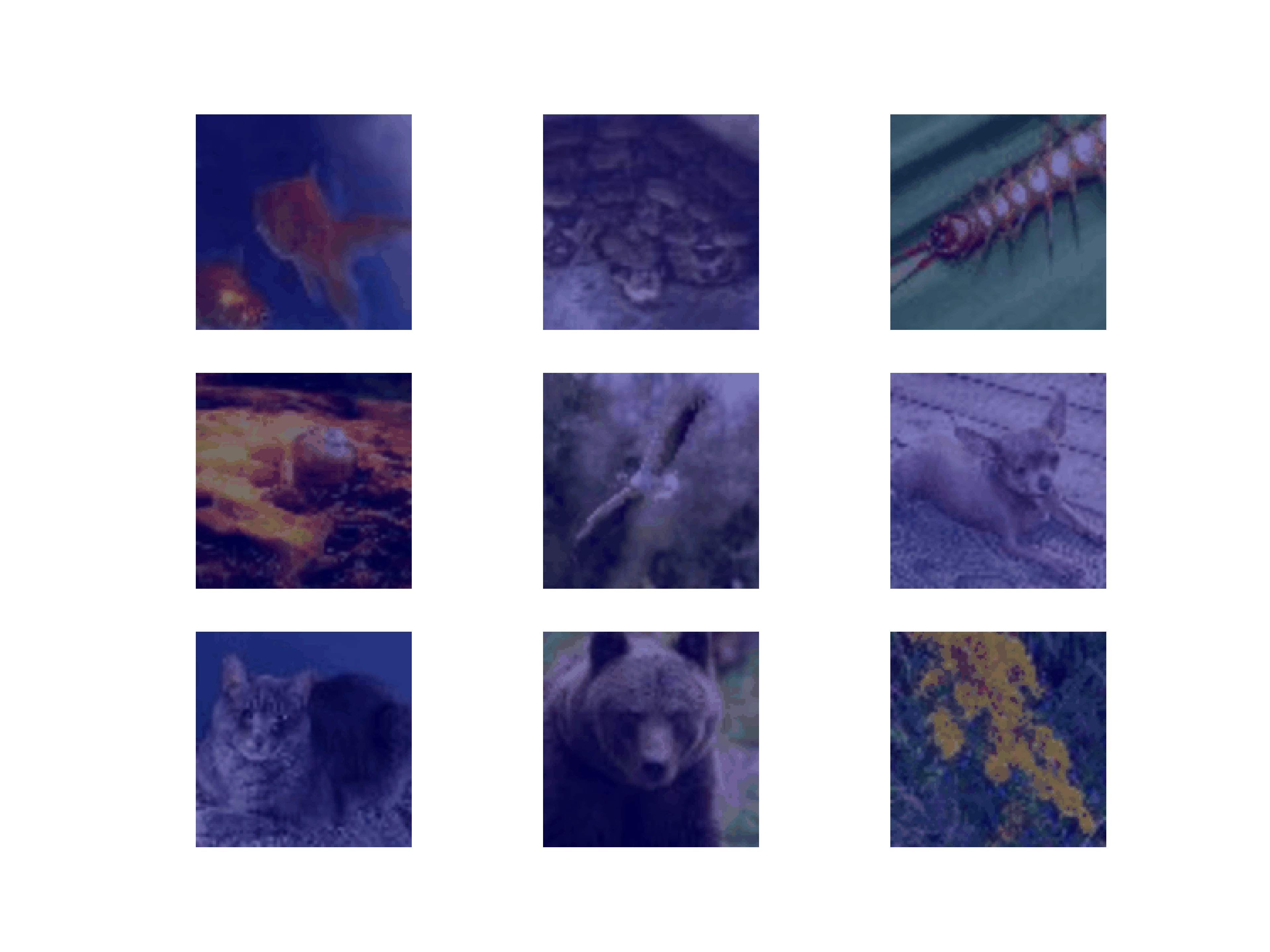

Supplement: Supplementary file 4 — Supplementary Information 4. [file 41598_2021_98448_MOESM4_ESM.gif]
