# Supplementary material for: Visual explanations from spiking neural networks using inter-spike intervals
Source: Sci Rep. 2021 Sep 24;11:19037. doi: 10.1038/s41598-021-98448-0 (PMC8463578; doi:10.1038/s41598-021-98448-0)
Supplement: Supplementary file 1 — Supplementary Information 1. [file 41598_2021_98448_MOESM1_ESM.pdf]

Supplementary Material for: Visual Explanations from Spiking Neural Networks using Inter-Spike Intervals

Youngeun Kim, Priyadarshini Panda
youngeun.kim@yale.edu, priya.panda@yale.edu

Department of Electrical Engineering, Yale University

Contents

Supplementary Figures

1) Spike activity analysis
2) SAM results of surrogate gradient learning (Layer 4)
3) SAM results of surrogate gradient learning (Layer6)
4) SAM results of surrogate gradient learning (Layer 8)
5) SAM results of ANN-SNN conversion (Layer 4)
6) SAM results of ANN-SNN conversion (Layer 6)
7) SAM results of ANN-SNN conversion (Layer 8)
8) SAM results of adversarial samples
9) The performance of SNN with respect to noisy images
10) Sensory suppression behavior in spiking neural networks

Supplementary Tables

1) Network architecture details

Supplementary Notes

1) Surrogate learning with BNTT
2) ANN-SNN conversion
3) Fast Gradient Sign Method Attack (FGSM)

Supplementary Figures

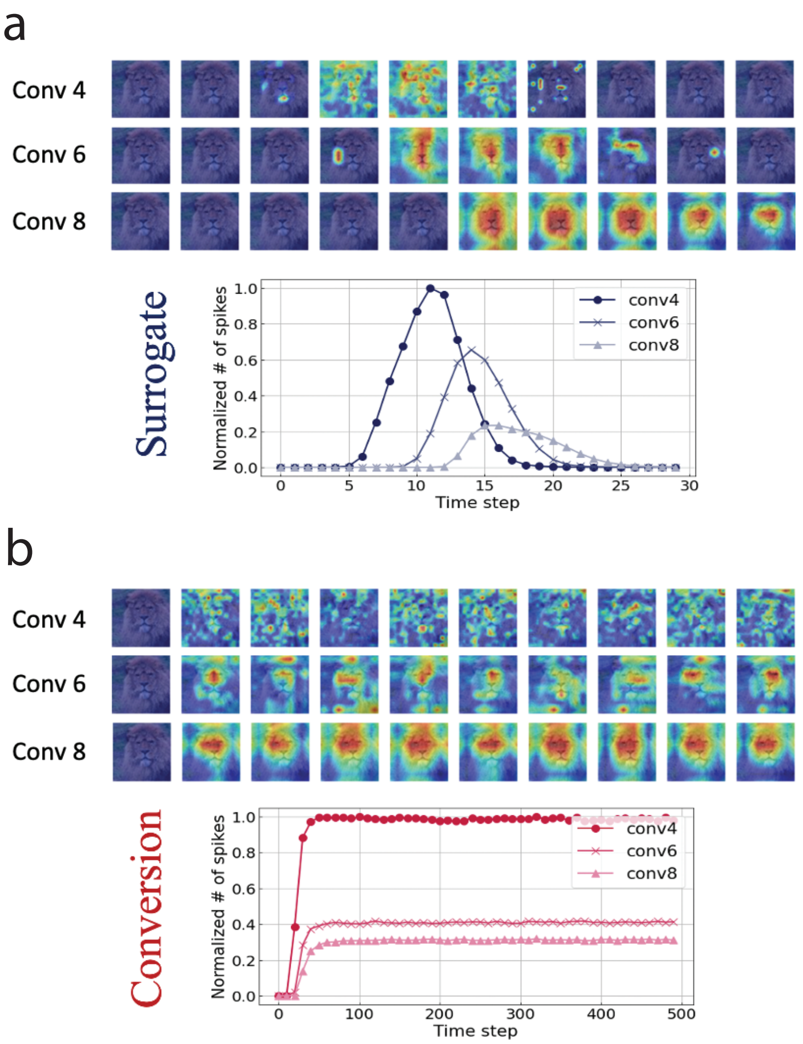

Supplementary Figure 1: Spike Activity Analysis. We observe that Spike Activation Map (SAM) visualizations obtained with surrogate gradient learning and conversion have different trends. (a) SAM with surrogate gradient learning shows Gaussian-like activity: zero value at initial time-steps, increasing activities in the middle, and diminishing at the end of the time-steps. This observation is caused by the temporal dynamics of spike activity integrated during surrogate gradient learning with BNTT (*i.e.*, SNN-crafted BN method). We see that the activity of the BNTT trained network also follows a gaussian-like profile. (b) On the other hand, the conversion method shows similar SAM across all time-steps. The conversion method does not show any temporal evolution in activity (mostly, constant after time-step$>$ 50) since the weights and spikes are derived from static ANNs. The results shown are for VGG11 architecture trained on Tiny-ImageNet dataset. For visualization, we uniformly sample 10 images across different time-steps.

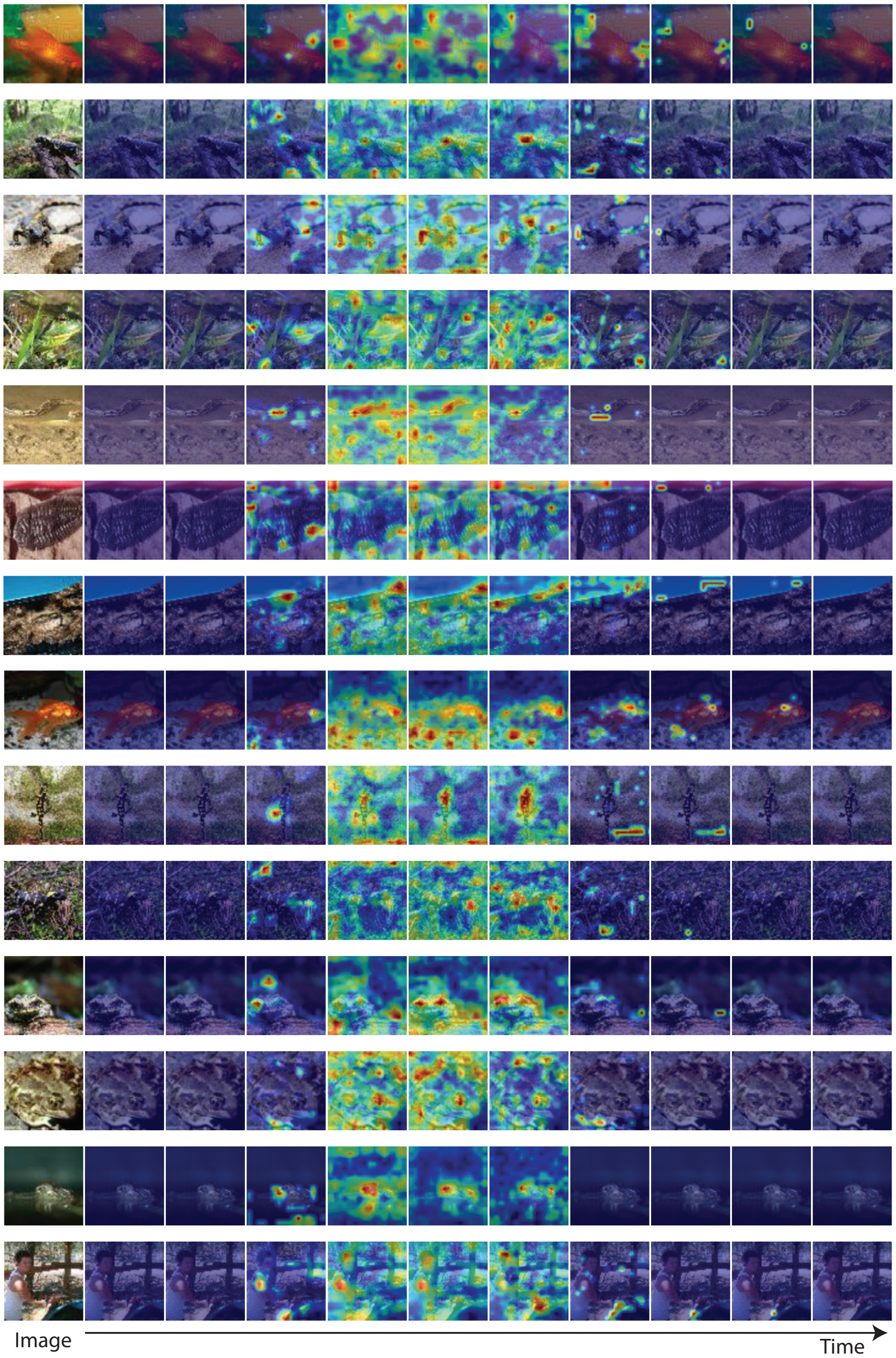

Supplementary Figure 2: SAM results of surrogate gradient learning at layer 4. We train VGG11 architecture on Tiny-ImageNet dataset for 30 time-steps. For visualization, we uniformly sample 10 images across timesteps. SAM in the shallow layer (*i.e.*, layer 4) highlights simple patterns such as edges and blobs.

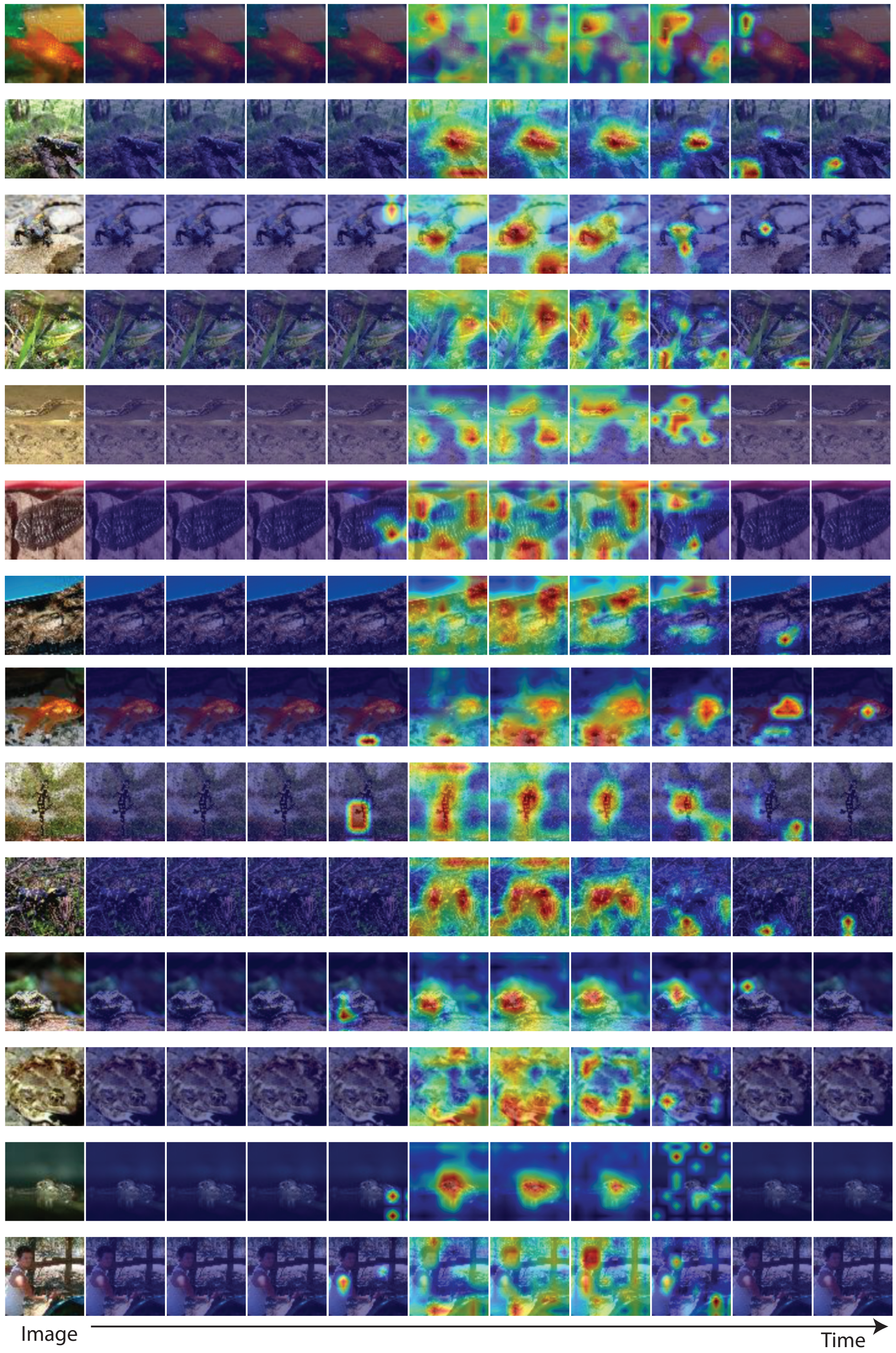

Supplementary Figure 3: SAM results of surrogate gradient learning at layer 6. We train VGG11 architecture on Tiny-ImageNet dataset with 30 time-steps. For visualization, we uniformly sample 10 images across timesteps. SAM in the intermediate layer (*i.e.*, layer 6) highlights shape-like information of the objects.

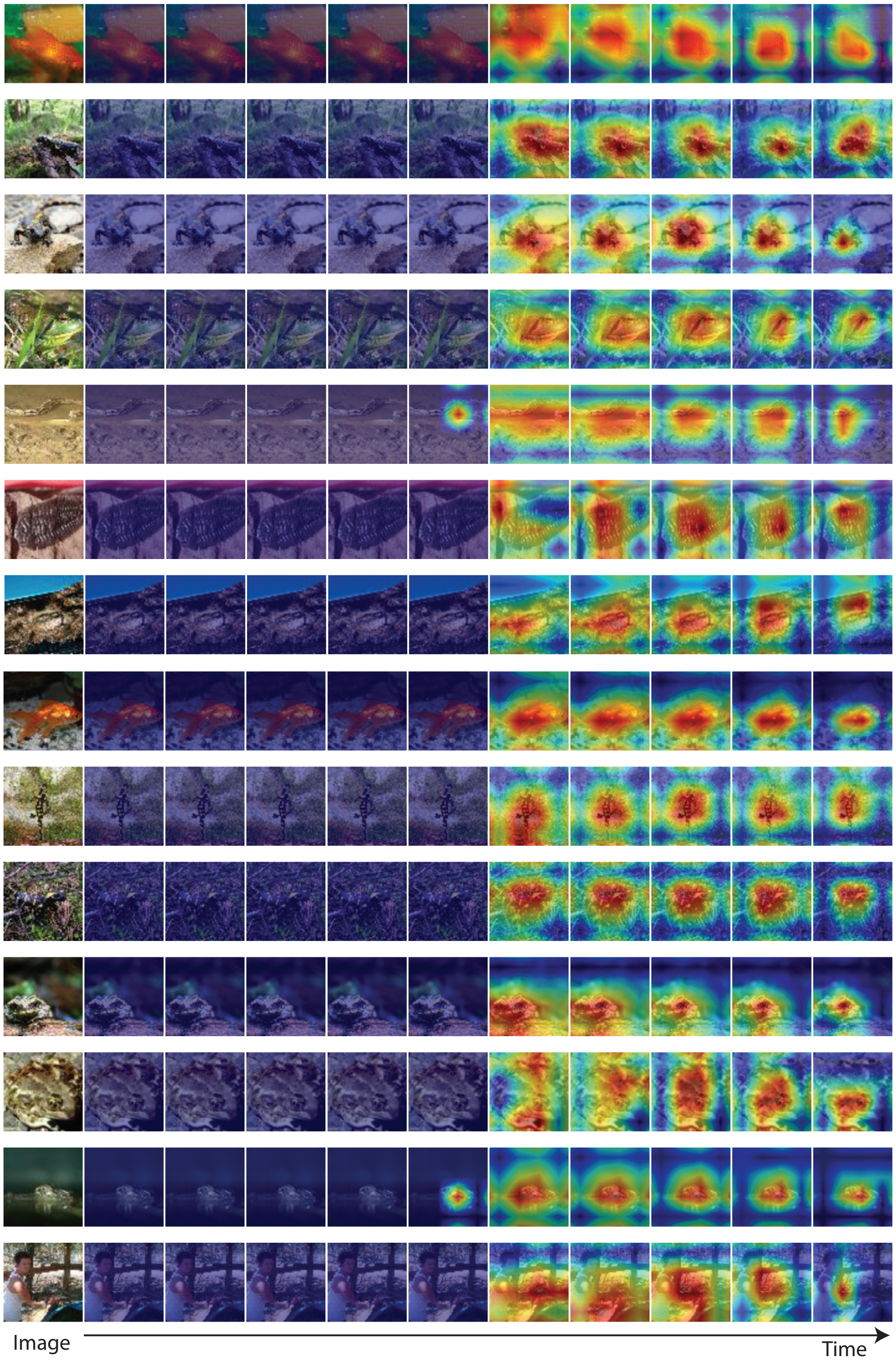

Supplementary Figure 4: SAM results of surrogate gradient learning at layer 8. We train VGG11 architecture on Tiny-ImageNet dataset with 30 time-steps. For visualization, we uniformly sample 10 images across timesteps. SAM in the deeper layer (*i.e.*, layer 8) highlights semantic information of the objects.

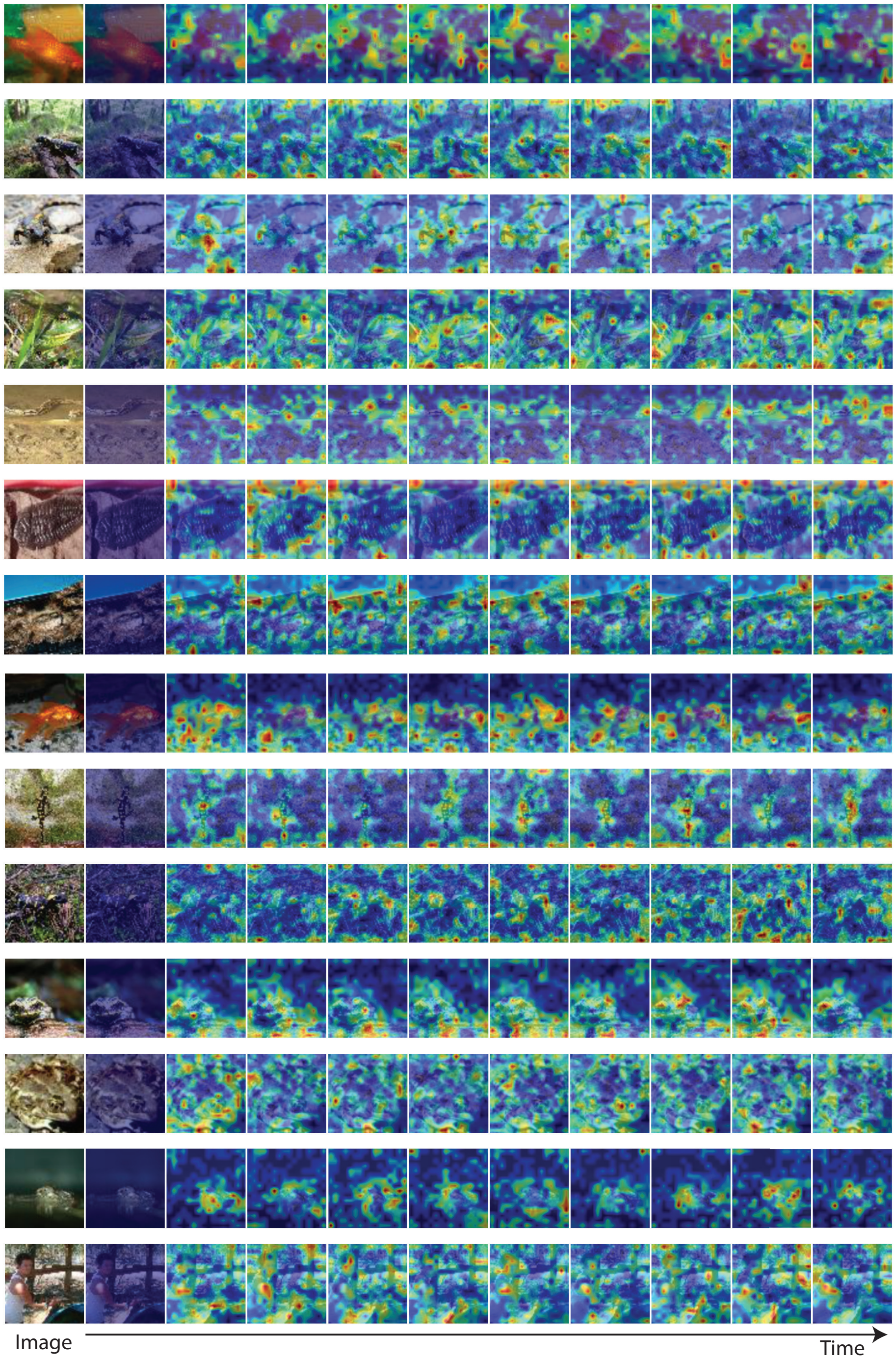

Supplementary Figure 5: SAM results of ANN-SNN conversion at layer 4. We convert a ReLU based VGG11 architecture trained on Tiny-ImageNet dataset with 500 time-steps. For visualization, we uniformly sample 10 images across timesteps.

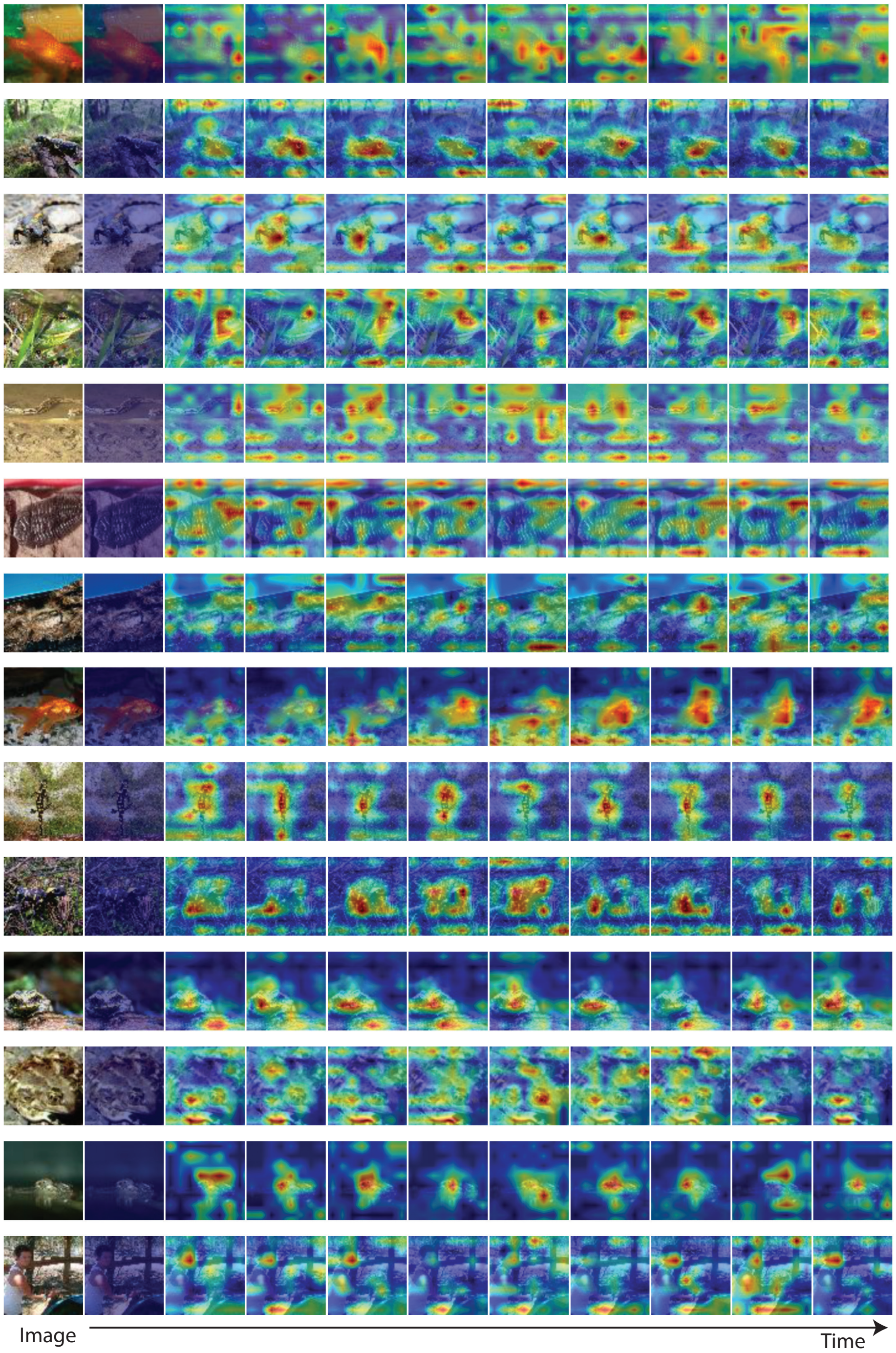

Supplementary Figure 6: SAM results of ANN-SNN conversion at layer 6. We convert VGG11 architecture trained on Tiny-ImageNet dataset with 500 time-steps. For visualization, we uniformly sample 10 images across timesteps.

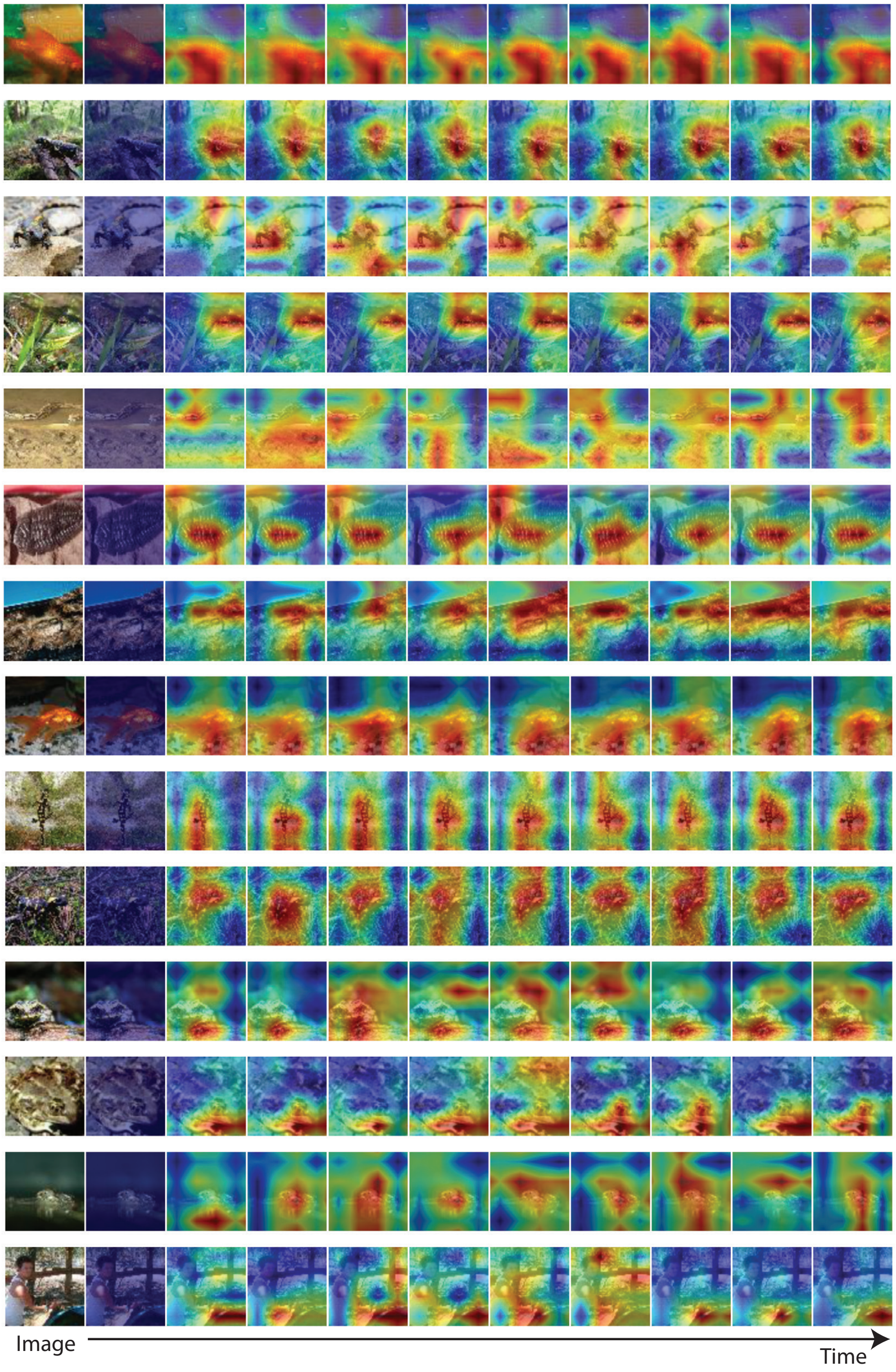

Supplementary Figure 7: SAM results of ANN-SNN conversion at layer 8. We convert VGG11 architecture trained on Tiny-ImageNet dataset with 500 time-steps. For visualization, we uniformly sample 10 images across timesteps.

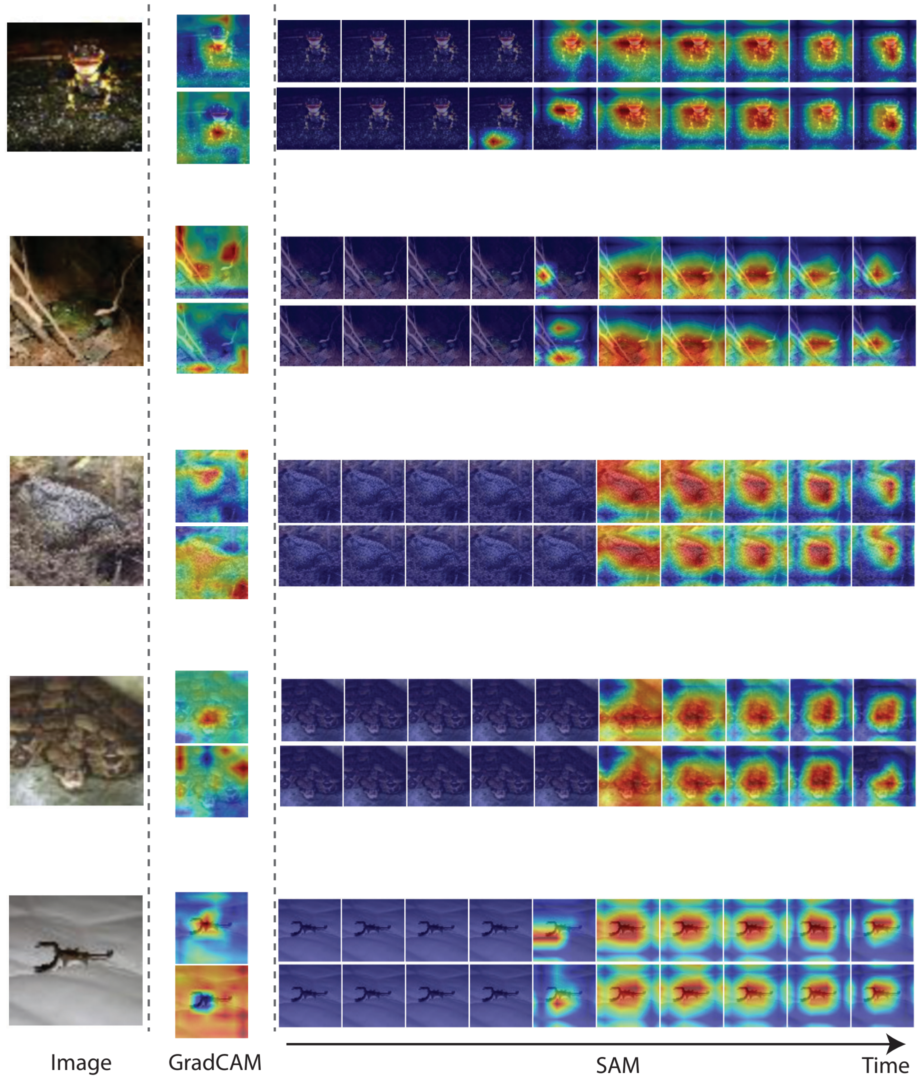

Supplementary Figure 8: SAM results of adversarial samples. We visualize Grad-CAM (with conventional ANN) and SAM (with surrogate gradient trained SNN) results. For each image , we show visualization for both clean image (top row) and adversarial image (bottom row). To generate adversarial image, we use Fast Gradient Sign Method (FGSM) attack with $\epsilon = \frac{4}{255}$. SAM visualization results show less difference between clean and adversarial images compared to Grad-CAM. Here, we use a VGG11 architecture trained on Tiny-ImageNet dataset.

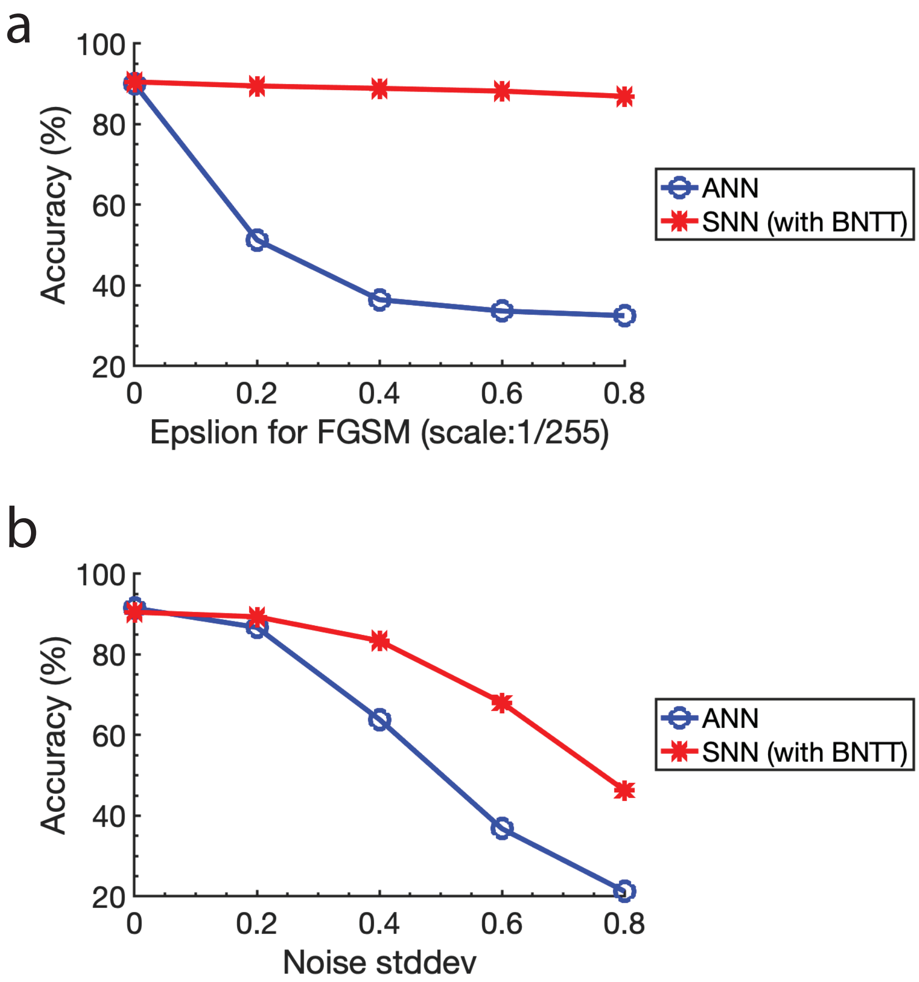

Supplementary Figure 9: The performance of SNN with respect to noisy images. To show the robustness of SNNs in terms of performance, we compare the accuracy of ANNs and SNNs with respect to adversarial and noisy samples. Here, we use a VGG9 architecture trained on CIFAR10 dataset. (a) We vary the intensity of FGSM attack (ϵ). The SNN with BNTT shows significant higher performance than the ANN counterpart across all noise configuration. (b) We add Gaussian noise to the image and show the performance by changing the standard deviation of the noise. We observe that SNN with BNTT achieve robust results across all noise configurations. Thus, SNNs are not only robust with respect to adversarial samples but also random noise environment.

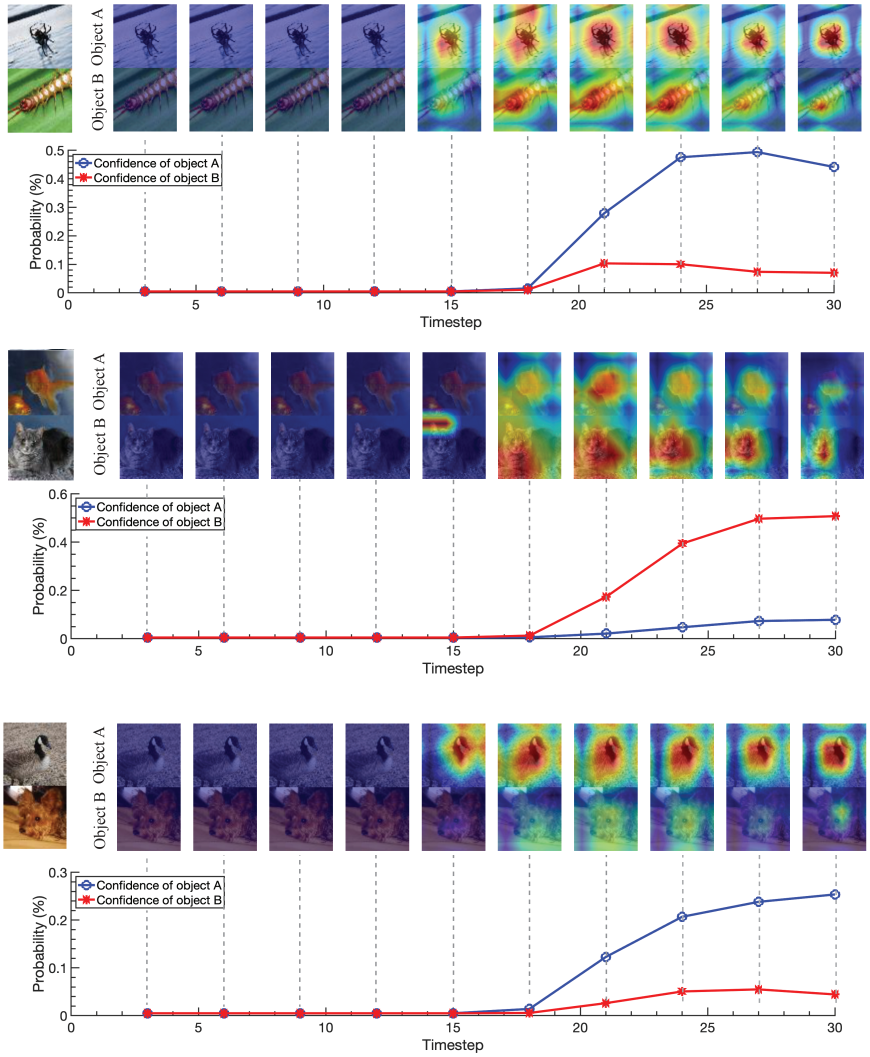

Supplementary Figure 9: Sensory suppression behavior in spiking neural networks. We concatenate two images vertically, and compute SAM of the image. For each timestep, we also visualize the probabilities of two classes in the last layer (*i.e.*, classifier). The results show that the network focuses on one of two objects. The confidence of each object is also varying according to the attended area by the network. The results shown are for VGG11 architecture trained on Tiny-ImageNet dataset with 30 time-steps. For visualization, we uniformly sample 10 images across time-steps.

Supplementary Tables

Supplementary Table 1: Network architecture details. We use a VGG11-like architecture for the experiments. Since we use an LIF neuron (or an IF neuron), we make a few changes on SNN architecture. Most importantly, we use average-pooling layers rather than max-pooling. This is because a max-pooling operation provides 1 as an output whenever at least one of the inputs has 1 (note, that SNNs convey information with 0 or 1 values), which causes information loss [3]. For surrogate gradient learning, we use SNN-crafted Batch Normalization Through Time (BNTT) technique proposed in [2]. For conversion method, we use dropout for training ANNs. Then, we convert trained ANNs to SNNs by using threshold balancing technique proposed in [5].

Layer type	Kernel size	Output channel	Stride
Conv	(3,3)	64	1
BNTT	-	64	1
Avgpool	(2,2)	-	2
Conv	(3,3)	128	1
BNTT	-	128	1
Avgpool	(2,2)	-	2
Conv	(3,3)	256	1
BNTT	-	256	1
Conv	(3,3)	256	1
BNTT	-	256	1
Avgpool	(2,2)	-	2
Conv	(3,3)	512	1
BNTT	-	512	1
Conv	(3,3)	512	1
BNTT	-	512	1
Avgpool	(2,2)	-	2
Conv	(3,3)	512	1
BNTT	-	512	1
Conv	(3,3)	512	1
BNTT	-	512	1
Avgpool	(2,2)	-	2
fc1	-	4096	-
BNTT	-	4096	-
fc2	-	200	-

Supplementary Notes

Algorithm 1 Surrogate Gradient Backpropagation

Input: Mini-batch (X); label set (Y); max_timestep (T)
Output: Updated network weights

for $i \leftarrow 1$ to max_iter **do**
    // fetch a mini batch X
    for $t \leftarrow 1$ to T **do**
        O $\leftarrow$ PoissonGenerator(X)
        for $l \leftarrow 1$ to $L-1$ **do**
            $(O_l^t, U_l^t) \leftarrow (\lambda, U_l^{t-1}, \mathrm{BNTT}_t(W_l, O_{l-1}^{t-1}))$
        end for
        $U_L^t \leftarrow (U_L^{t-1}, \mathrm{BNTT}_t(W_L, O_{L-1}^{t-1}))$ ▷ for final layer, stack the voltage
    end for
    $L \leftarrow (U_L^T, Y)$ ▷ Calculate the loss and back-propagation
end for

Algorithm 2 ANN-SNN Conversion

Input: Input set (X); label set (Y); max_timestep (T); pre-trained ANN model (A); SNN model (S); total layer number (L)
Output: Updated SNN network with threshold balancing

$S.weights \leftarrow A.weights$ ▷ Copy ANN weights to SNN
$S.th \leftarrow 0$ ▷ Initialize threshold voltage
for $l \leftarrow 1$ to $L-1$ **do**
    for $t \leftarrow 1$ to T **do**
        $O^t \leftarrow$ PoissonGenerator(X)
        for $l_{tmp} \leftarrow 1$ to l **do**
            if $l_{tmp} < l$ **then**
                $(O_l^t, U_l^t) \leftarrow (U_l^{t-1}, W_l, O_{l-1}^{t-1})$
            else
                $S_l.th \leftarrow \max(S_l.th,\ W_l O_{l-1}^{t-1})$ ▷ Threshold update for each layer
            end if
        end for
    end for
end for

Algorithm 3 SNN-crafted FGSM attack

Input: target class (c); attack intensity (ϵ); SNN model (SNN); spike time-step (T)
Output: class representative image x

```
Fetch input image x                         ▷ Fetching input
W_1 ← SNN.conv1.weight                      ▷ First convolution weight
G = 0                                       ▷ Accumulated gradient at the first conv layer
for t ← 1 to T do
    y ← SNN(x_t)
    G+ = (1/T) ∂y_c/∂x_conv1                ▷ Accumulate gradients in layer 1
end for
Δx ← Deconvolution(W_1, G)                  ▷ Deconvolution
x_adv ← x + ε × sign(Δx)
```

1 Surrogate Learning with BNTT

A suitable backpropagation process is especially important in SNNs since the functionality of an LIF neuron is non-differentiable. In [4, 7], the authors showed that by using recurrent flow for computational graphs, we can train SNNs with accumulated spike and gradient information. The accumulated gradients at the hidden layer and the output layer can be represented as:

$$\Delta W_l = \frac{\partial L}{\partial W_l} = \begin{cases} \sum_t \left(\frac{\partial L}{\partial O_l^t} \frac{\partial O_l^t}{\partial U_l^t} + \frac{\partial L}{\partial U_l^{t+1}} \frac{\partial U_l^{t+1}}{\partial U_l^t} \right) \frac{\partial U_l^t}{\partial W_l}, & \text{if } l : \text{hidden} \\ \frac{\partial L}{\partial U_l^T} \frac{\partial U_l^T}{\partial W_l}. & \text{if } l : \text{output} \end{cases} \tag{1}$$

where, W_l, O_l and U_l stand for weight matrix, output spike matrix, and membrane potential matrix at layer l, respectively. As the output layer does not generate spikes, we compute the exact derivative of the loss L with respect to the membrane potential u_i^T:

$$\frac{\partial L}{\partial u_i^T} = \frac{e^{u_i^T}}{\sum_{k=1}^{C} e^{u_k^T}} - y_i. \tag{2}$$

However, for hidden layer, the gradient term $\frac{\partial o_i^t}{\partial u_i^t}$ is not differentiable due to the firing behavior of an LIF neuron. Therefore, $\frac{\partial o_i^t}{\partial u_i^t}$ should be formulated with an approximated continuous function. To this end, we use a piecewise linear function:

$$\frac{\partial o_i^t}{\partial u_i^t} = \beta \max\{0, 1 - |\frac{u_i^t - \theta}{\theta}|\}, \tag{3}$$

where, β is a scaling factor for the gradient value. We set β as 0.3 to prevent a gradient exploding problem. Based on the gradient value, the weights of SNNs are updated. Algorithm 1 summarizes the whole training process of surrogate gradient training.

2 ANN-SNN Conversion

We use [5] for implementing the ANN-SNN conversion method. We use the threshold balancing method to perform the conversion. The overall algorithm for the conversion method is shown in Algorithm 2.

3 Fast Gradient Sign Attack Method (FGSM)

In order to further validate the robustness of spiking neural networks with the proposed SAM, we conduct experiments on adversarial inputs. We use Fast Gradient Sign Method (FGSM) attack [1] to generate adversarial samples for ANN. For a given image x, we compute the loss function $\mathcal{L}(x, y)$ with the ground truth label y. The objective of FGSM attack is to change the pixel intensity of the input image that maximizes the cost function:

$$x_{adv} = x + \epsilon \times sign(\nabla_x \mathcal{L}(x, y)). \tag{4}$$

We call x_{adv} as "adversarial sample". Here, ϵ denotes the strength of the attack. To conduct the FGSM attack for SNN, we use the SNN-crafted FGSM method proposed in [6]. Since SNNs process information temporally, the authors of [6] accumulate the gradients across the time axis. According to the sign of accumulated gradients, they generate a noise for the adversarial sample. We summarize the overall process of SNN-crafted FGSM in Algorithm 3.

References

[1] Ian J Goodfellow, Jonathon Shlens, and Christian Szegedy. Explaining and harnessing adversarial examples. *arXiv preprint arXiv:1412.6572*, 2014.

[2] Youngeun Kim and Priyadarshini Panda. Revisiting batch normalization for training low-latency deep spiking neural networks from scratch. *arXiv preprint arXiv:2010.01729*, 2020.

[3] Chankyu Lee, Syed Shakib Sarwar, Priyadarshini Panda, Gopalakrishnan Srinivasan, and Kaushik Roy. Enabling spike-based backpropagation for training deep neural network architectures. *Frontiers in Neuroscience*, 14, 2020.

[4] Emre O Neftci, Hesham Mostafa, and Friedemann Zenke. Surrogate gradient learning in spiking neural networks. *IEEE Signal Processing Magazine*, 36:61–63, 2019.

[5] Abhronil Sengupta, Yuting Ye, Robert Wang, Chiao Liu, and Kaushik Roy. Going deeper in spiking neural networks: Vgg and residual architectures. *Frontiers in neuroscience*, 13:95, 2019.

[6] Saima Sharmin, Nitin Rathi, Priyadarshini Panda, and Kaushik Roy. Inherent adversarial robustness of deep spiking neural networks: Effects of discrete input encoding and non-linear activations. *arXiv preprint arXiv:2003.10399*, 2020.

[7] Yujie Wu, Lei Deng, Guoqi Li, Jun Zhu, and Luping Shi. Spatio-temporal backpropagation for training high-performance spiking neural networks. *Frontiers in neuroscience*, 12:331, 2018.
